# Healthcare utilisation and economic burden of migraines among bank employees in China: a probabilistic modelling study

**DOI:** 10.1186/s10194-024-01763-w

**Published:** 2024-04-19

**Authors:** Du Wei, Li Ping Wong, Xun He, Tharani Loganathan

**Affiliations:** 1 School of Medicine and Health Management, Guizhou Medical University, Guiyang, China; 2 Department of Social and Preventive Medicine, Faculty of Medicine, Universiti Malaya, Kuala Lumpur, Malaysia; 3 Center of Medicine Economics and Management Research, Guizhou Medical University, Guiyang, China; 4 Centre for Epidemiology and Evidence-Based Practice, Department of Social and Preventive Medicine, Universiti Malaya, Kuala Lumpur, Malaysia

**Keywords:** Migraine disorder, Cost of illness, Healthcare utilisation

## Abstract

**Background:**

Despite the recognised high prevalence of migraines among bank employees, yet their healthcare utilisation patterns and the economic burden of migraines remain underexplored.

**Aim:**

To examine migraine-related healthcare utilisation among bank employees in China, and to estimate the economic burden of migraines.

**Methods:**

A cross-sectional survey was conducted in Guizhou province, China between May and October 2022. The HARDSHIP questionnaire was used to identify migraine-positive individuals and enquire about their healthcare utilisation and productivity losses. A probabilistic decision-analytic model with a micro-costing approach was used to estimate the economic burden from the perspectives of the healthcare system, employers, and society. All costs were expressed in 2022 United States dollars. One-way and probabilistic sensitivity analyses were performed.

**Results:**

Nearly half of individuals with migraines reported not seeking medical care. Only 21.8% reported seeking outpatient consultations, 52.5% reported taking medicines, and 27.1% reported using complementary therapies. Chronic migraine patients had significantly higher healthcare utilisation than episodic migraine patients. Among individuals with a monthly migraine frequency of 15 days or more, 63.6% took inappropriate treatments by excessively using acute medications. Migraines in the banking sector in Guizhou cost the healthcare system a median of $7,578.0 thousand (25th to 75th percentile $4,509.2–$16,434.9 thousand) per year, employers $89,750.3 thousand (25th to 75th percentile $53,211.6–$151,162.2 thousand), and society $108,850.3 thousand (25th to 75th percentile $67,370.1–$181,048.6 thousand). The median societal cost per patient-year is $3,078.1. Migraine prevalence and productivity losses were identified as key cost drivers.

**Conclusions:**

The study points to the need to raise awareness of migraines across all stakeholders and to improve the organisation of the migraine care system. A substantial economic burden of migraines on the healthcare system, employers, and society at large was highlighted. These cost estimates offer evidence-based benchmarks for assessing economic savings from improved migraine management, and can also draw the attention of Chinese policymakers to prioritise migraine policies within the banking and other office-based occupations.

**Supplementary Information:**

The online version contains supplementary material available at 10.1186/s10194-024-01763-w.

## Introduction

Migraines stand as a significant and pressing public health concern [1–3], contributing to a substantial number of years lived with disability worldwide. This illness has consistently been identified in Global Burden of Disease studies as a major contributor to disability-related disease burden [4, 5]. Beyond the disabling effects of migraines, there is compelling evidence indicating that migraine elevates the risk of stroke and cardiovascular diseases [6, 7].

However, despite the well-documented impacts of migraines, the issues of underdiagnosis and undertreatment remain widespread. This challenge is not exclusive to low- and middle-income countries, where resources are limited and healthcare access is often inadequate, it also prevails in high-income countries, including those in Europe and North America [8]. A concerning revelation is that more than half of individuals suffering from migraines do not seek a medical diagnosis for their conditions [9]. Furthermore, an even smaller proportion of them receive adequate treatment. This treatment gap is evident globally, highlighted by a survey finding showing that only 49% of migraine sufferers in Germany receive adequate care [10], and in China, the use of triptans—a migraine-specific medication [2]—is almost non-existent [9].

Recent research has unveiled a notable prevalence of migraines among banking employees in China [11], aligning with findings from studies in various other countries [12, 13]. This prevalence is considerably higher than that observed in the general Chinese population [11]. Identified risk factors for migraines among bank employees include office environments [14], sedentary behaviours [15, 16], forward head postures [17] and excessive job pressure [18, 19]. While there has been recognition of the heightened prevalence of migraines in the banking sector, there remains a significant gap in publications examining the patterns of healthcare utilisation among bank employees suffering from migraines. This underscores the importance of investigating and understanding the healthcare-seeking behaviours of this specific population to address the existing knowledge gap.

Migraines place a considerable economic burden on healthcare systems, employers, and society. Yet, there are no studies from China that report the per-patient costs from the three perspectives. Referencing examples from other countries, the annual cost per migraine patient to the healthcare system is $1,066.0 in Europe [20] and $7,578.1 in the United States (U.S.) [21]. All costs reviewed in the study have been converted to 2022 United States dollars (USD) using Gross Domestic Product (GDP) deflator indexes and purchasing power parity values, for comparison purposes. From the employers’ perspective, in the U.S., employees with migraines cost employers an additional $2,708.5 per patient-year [21]. Societally, the annual cost per patient amounts to $10,286.6 in the U.S. [21] and $15,148.2 in Europe [20]. Despite the well-established economic impacts, the specific costs of migraines among banking employees in China, a population with a notably high prevalence of migraines, remain undocumented. Capturing these costs within the specific population is vital, as it offers important evidence-based insights for policymakers to guide prioritisation, shape policy development, and allocate health funds, particularly amidst resource constraints.

This study aims to examine migraine-related healthcare utilisation patterns and to estimate the economic burden of migraines among bank employees in China.

## Methods

### Study overview

**Fig. 1:**
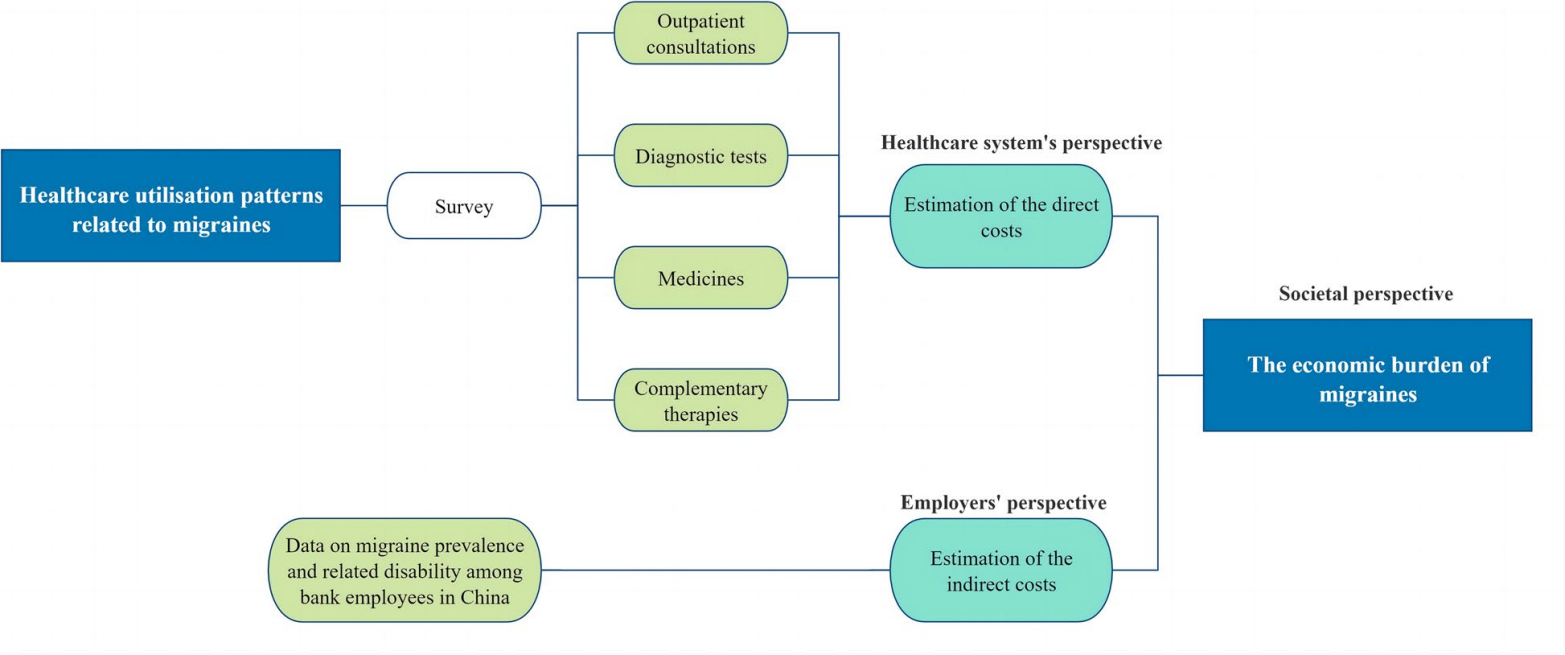
Research framework of this study

The data analysed in this study were derived from a cross-sectional survey of bank employees in Guizhou province in China, conducted between May and October 2022 [11]. Ethical approval for this research was obtained from the Research Ethics Committee of Guizhou Medical University in China (Approval number 2021251). The selection of bank employees was performed using probability sampling methods. Further details regarding the survey methodology (including study setting, participant eligibility, sample size and selection, and participant engagement), data processing, and participant characteristics are available in our previously published paper [11]. Our study achieved a response rate of 97.2%, thus indicating that we obtained a highly representative sample of bank employees. Figure 1 illustrates the research framework for this study.

The HARDSHIP questionnaire, which was developed based on the third beta edition of the International Classification of Headache Disorders [22], was employed for migraine diagnosis in our survey. This instrument has been demonstrated to be ideal for diagnosing migraines in non-clinical settings across diverse cultures [23] and has been validated in the Chinese language [24]. Our survey findings indicated that 525 out of 1,929 employees were diagnosed with migraines, resulting in a prevalence rate of 27.2% (95% CI 25.2–29.2%) [11].

In line with previous studies on healthcare utilisation patterns among Chinese individuals with migraines [25–28], our survey specifically targeted the utilisation patterns of outpatient consultations, diagnostic tests, medicines, and complementary therapies related to migraines among bank employees. Hospitalisations and emergency room visits were not considered in this study, as previous research has shown that these services are rarely used by migraine patients in China [25–27].

Moreover, using the data on healthcare utilisation, it was possible to estimate the direct medical costs of migraines. Simultaneously, using the data on migraine prevalence and related disability among bank employees in China, as outlined in our previous paper [11], enabled the estimation of the indirect costs of migraines.

Accordingly, the economic burden of migraines was estimated from the perspectives of the healthcare system, employers, and society. From the healthcare system’s perspective, direct medical costs were calculated, encompassing costs related to outpatient consultations, diagnostic tests, medicines, and complementary therapies. Direct non-medical costs were not considered, given that migraine sufferers rarely utilise special transportation or social services for healthcare seeking [20]. From the employers’ perspective, indirect costs were estimated. The combination of both direct and indirect costs provided a holistic societal perspective on the economic burden of migraines.

The costs reported in this study are presented in 2022 prices and are denominated in USD, adjusted using GDP deflator indexes (sourced from the World Bank database) and purchasing power parity values (sourced from the Organisation for Economic Co-operation and Development database). The reporting of this study adhered to the guidelines outlined in the Consolidated Health Economic Evaluation Reporting Standards 2022 (CHEERS 2022) statement [29] and referred to the checklists specifically tailored for micro-costing studies [30].

### Healthcare utilisation related to migraines

The HARDSHIP questionnaire incorporates a module on healthcare utilisation. However, since the questions and response options within this questionnaire may differ depending on the country setting, World Health Organization (WHO) experts recommend adapting the questionnaire to align with the specific healthcare system and country context [31].

The adapted HARDSHIP healthcare utilisation questionnaire is provided in Supplementary Material 1. Prior to its application, the questionnaire underwent a cross-cultural validation to ensure its validity for the target population. The details of the cross-cultural validation process are further elucidated in Supplementary Material 1.

Migraines were categorised based on their frequency, with those occurring 15 days or more per month classified as chronic migraines (CM) and those occurring less than 15 days per month as episodic migraines (EM). Categorial data were presented by frequency (N) and percentage (%), while continuous data were presented by mean and standard deviation (SD), as well as median and range. The utilisation of healthcare resources between EM and CM patients was compared using the Chi-square test (or Fisher’s exact test) for categorical data and using the Mann–Whitney *U* test for continuous data. A *p*-value < 0.05 was considered as statistically significant. All statistical analyses were performed using SPSS software version 26 (IBM Corporation, Armonk, NY, U.S.).

### Economic burden of migraines

#### Decision-analytic modelling

**Fig. 2:**
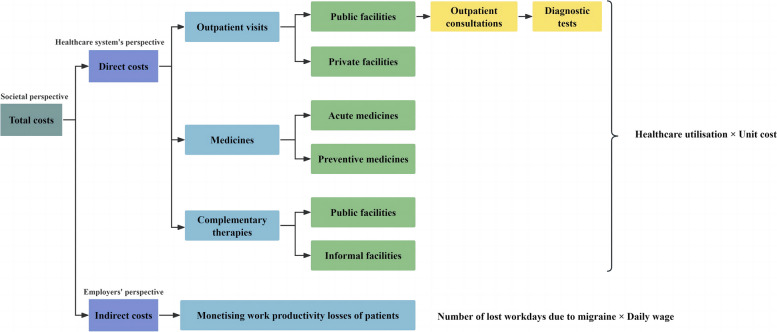
Decision-analytic model for the economic burden of migraines Note: This study simulated patients’ clinical pathways, considering outpatient visits at both public and private facilities. Since the unit costs of health services in China are available from an official tariff exclusively for public facilities, the study focused on detailing the outpatient visit pathway within these public healthcare settings. Typically, this pathway begins with an outpatient consultation, followed by diagnostic tests. The costs of these two components were aggregated to calculate the costs for outpatient visits at public facilities. Regarding outpatient visits at private facilities, there is no officially standardised tariff for healthcare services in China, as the pricing in these facilities is influenced by market forces. Nevertheless, the National Health Commission of the People’s Republic of China annually compiles an all-cause average fee per outpatient visit, encompassing outpatient consultations and diagnostic tests, across all providers [34]. This average fee per outpatient visit could be used to calculate the costs for outpatient visits at private facilities This model’s validation process included evaluations of face validity and internal validity, taking into account its realisability. Following established guidelines for model validation [32], the evaluation of face validity was carried out by the six experts mentioned in Supplementary Material 1, and the evaluation of internal validity was carried out by our research team

A probabilistic decision-analytic model was developed to estimate the economic burden of migraines, using a micro-costing approach and adopting the perspectives of the healthcare system, employers, and society (Fig. 2). The model was validated for face validity and internal validity, following established guidelines for model validation [32].

Given the chronic nature of migraine disease, a prevalence-based method was more suitable for studying its burden than an incidence-based method. This led to the adoption of a one-year time horizon for this model. The bottom-up approach was employed to estimate direct costs, while the human capital approach was employed to estimate indirect costs.

From the health system’s perspective, the model captured the current treatment practices for migraines in China, as previously reviewed. This includes outpatient consultations, diagnostic tests, medicines, and complementary therapies [25–28], enabling the estimation of direct medical costs. From the employers’ perspective, indirect costs were calculated by assigning monetary values to the decrease in work productivity experienced by individuals with migraines. From the societal perspective, the economic burden was the sum of direct and indirect costs attributed to migraines [33], irrespective of who bears these costs.

The data sources for the cost estimation were selected with caution, as it is important to mention that the data sources of a Cost-of-Illness (COI) study are related to the decision-making context, which is characterised by the jurisdiction, healthcare system, and population [35]. Given the jurisdiction and healthcare system in Guizhou province of China, this study employed the 2022 healthcare services tariff of Guizhou province, the statistical communiqué of the People’s Republic of China on the 2022 national healthcare development, and a publicly available source “Medicine Price Checker” to determine unit costs of healthcare resources. Considering the specific population of bank employees in this model, our survey data served as the primary source to offer the best evidence on prevalence, healthcare utilisation, and productivity losses, rather than existing literature. The cost estimation in this study was conducted on an annual basis, hence the application of a discount rate was not necessary.

#### Number of migraine sufferers

This study calculated the number of migraine sufferers by gender using Eq. (1):

1 $$\begin{array}{c}Number\;of\;migraine\;sufferers\;by\;gender\\=Population\;size\\\times\;Proportion\;of\;males\;or\;females\\\times\;Prevalence\;rate\;of\;migraines\;by\;gender\end{array}$$

Due to the unavailability of specific statistics on the population size of banking employees in China, employees in the financial sector were taken as a proxy population in this study. According to the Guizhou Provincial Bureau of Statistics [36], the financial sector employed 141.8 thousand individuals in 2022, with a male-to-female ratio of 50.4% to 49.6%. The prevalence of migraines, as reported in our previous publication [11], was 25.0% among males and 29.2% among females.

#### Direct costs

According to the decision-analytic model depicted in Fig. 2, individuals receiving migraine treatment may have costs for outpatient visits, medicines, and complementary therapies. The direct costs were calculated using Eq. (2):

2 $$\begin{array}{c}Direct\;costs\\=Costs\;for\;outpatient\;visits\\+\;Costs\;for\;medicines\\+\;Costs\;for\;complementary\;therapies\end{array}$$

Among these, the costs for outpatient visits encompass costs for outpatient consultations and diagnostic tests. These costs were determined by multiplying the respective unit costs in 2022 by the estimated annual utilisation of migraine-related healthcare services, as illustrated in Eq. (3). The specific unit costs were estimated and can be found in Supplementary Material 2. Meanwhile, the annual estimates for migraine-related utilisation of outpatient consultations and diagnostic tests were calculated using our survey data.

3 $$\begin{array}{c}Costs\;for\;outpatient\;consultations\;or\;diagnostic\;tests\\=Unit\;cost\;of\;a\;consultation/test\\\times\;Annual\;number\;of\;consultations/tests\end{array}$$

As depicted in the decision-analytic model in Fig. 2, this study simulated patients’ clinical pathways, considering outpatient visits at both public and private facilities. Since the unit costs of health services in China are available from an official tariff exclusively for public facilities, the study focused on detailing the outpatient visit pathway within these public healthcare settings. Typically, this pathway begins with an outpatient consultation, followed by diagnostic tests. The costs of these two components were aggregated to calculate the costs of outpatient visits at public facilities. Regarding outpatient visits at private facilities, there is no officially standardised tariff for healthcare services in China, as the pricing in these facilities is influenced by market forces. Nevertheless, the National Health Commission of the People’s Republic of China annually compiles an all-cause average fee per outpatient visit, encompassing outpatient consultations and diagnostic tests, across all providers [34]. This average fee per outpatient visit could be used to calculate the costs for outpatient visits at private facilities.

To estimate the costs for medicines, the annual number of medication days for each medicine was multiplied by the corresponding daily cost, as illustrated in Eq. (4). This calculation aligns with the HARDSHIP healthcare utilisation questionnaire, which simplifies questions for respondents by focusing on the number of medication days instead of detailed dosage specifics. The annual number of medication days was determined from our survey data, while the daily costs of these medicines were estimated and are provided in Supplementary Material 2.

4 $$\begin{array}{c}Costs\;by\;medicine\;type\\=Daily\;cost\;by\;medicine\;type\\\times\;Annual\;medication\;days\;by\;medicine\;type\end{array}$$

For the costs of complementary therapies, owing to the unstandardised nature of complementary therapies and the personalised treatment regimens administered to individual patients, estimating the precise costs of a single session of these therapies poses a challenge. Consequently, in our survey, participants were queried about the expenses they paid for each type of migraine-related complementary therapy in 2022. The per-patient costs by therapy and facility type (refer to Supplementary Material 2) were then multiplied by the corresponding estimated annual number of patients receiving complementary therapies to determine the overall costs of complementary therapies, as illustrated in Eq. (5):

5 $$\begin{array}{c}Costs\;for\;complementary\;therapies\\=\;Per-patient\;cost\;for\;complementary\;therapies\;in\;2022\\\times\;Annual\;number\;of\;patients\;receiving\;these\;therapies\end{array}$$

#### Indirect costs

The indirect costs were calculated by gender using Eq. (6):

6 $$\begin{array}{c}Indirect\;costs\;by\;gender\\=Number\;of\;migraine\;sufferers\;by\;gender\\\times\;Daily\;wage\;by\;gender\\\times Annual\;lost\;work\;days\;for\;a\;patient\;due\;to\;migraine\;by\;gender\end{array}$$

The combined indirect costs of migraines for males and females were the total indirect costs. The number of migraine sufferers by gender was calculated based on the respective prevalence rates obtained from our survey data. Daily wages by gender were computed from our survey data by dividing the median monthly wages, including take-home pay, benefits, and payroll tax [33, 35], by 22 (assuming 22 working days per month). The resulting estimated daily wages for males and females were both $83.9 in 2022 USD. To estimate the annual lost workdays for an individual due to migraine, we utilised the gender-specific number of working days lost over a three-month period, as derived from our survey. The lost workdays for both males and females were then extrapolated to a full year by multiplying by four.

#### Point estimation and sensitivity analyses

Supplementary Material 3 provides a list of model inputs and data sources for estimating the annual costs of migraines. Initially, the point estimation was performed based on the decision-analytic model illustrated in Fig. 2. In cases where input parameters exhibited skewed distributions, median values were employed in this analysis.

Subsequently, to determine which parameter had the greatest impact on the total costs, a one-way sensitivity analysis was performed by varying each key parameter at a time over a ± 20% variation range. A tornado diagram was used to show the results.

Finally, the joint uncertainty across all input parameters was evaluated by probabilistic sensitivity analyses. How these parameters were incorporated into the probabilistic decision-analytic model is also detailed in Supplementary Material 3. This sensitivity analyses involved 10,000 Monte Carlo simulations for input parameters incorporated as probability distributions by using R software version 4.1.3 (R Foundation, a non-profit organisation).

## Results

### Characteristics of individuals diagnosed with migraines

Of the 525 individuals diagnosed with migraines, 41.6% were male, and 58.4% were female. Based on the frequency of migraines, 466 (88.8%) respondents were categorised as having EM, while 59 (11.2%) respondents as having CM.

### Migraine-related healthcare utilisation

Table 1 shows data on the annual healthcare service utilisation related to migraines among the surveyed individuals, and Table 2 shows their medicine usage patterns. The most common outpatient consultation facility was public clinics, utilised by 8.7% of the respondents. Individuals diagnosed with CM had a significantly greater likelihood of seeking outpatient consultations at public tertiary-level hospitals, as compared to those diagnosed with EM (11.8% for CM vs. 3.8% for EM, *p* < 0.05).

**Table 1:** Utilisation of healthcare services for migraines within one year prior to survey completion

Healthcare resource	All patients N (%)	EM (*N* = 466)			CM (*N* = 59)^a^		
		**%**	**Mean visits/tests (SD)**	**Median (range)**	**%**	**Mean visits/tests (SD)**	**Median (range)**
**Outpatient consultations**							
Public clinics	46 (8.7)	8.6	2.9 (1.6)	2.0 (1–6)	9.6	1.8 (0.5)	2.0 (1–2)
Public primary-level hospitals	25 (4.7)	4.8	3.2 (2.2)	2.1 (1–9)	3.9	3.9 (2.7)	3.8 (2–6)
Public secondary-level hospitals	35 (6.6)	6.3	2.8 (2.0)	2.0 (1–8)	9.4	4.1 (3.0)	3.0 (1–9)
Public tertiary-level hospitals	25 (4.7)	3.8	1.8 (0.7)	2.0 (1–3)	11.8*	2.0 (0.6)	2.0 (1–3)
Public TCM hospitals	19 (3.6)	2.9	3.9 (2.9)	3.0 (1–9)	8.8	2.6 (1.5)	2.1 (1–5)
Private facilities	11 (2.1)	1.8	4.5 (2.3)	5.0 (1–8)	4.8	6.2 (1.8)	5.6 (5–8)
**At any facilities**	**114 (21.8)**	**21.1**	N/A	N/A	**27.2**	N/A	N/A
**Diagnostic tests**							
CT scan	57 (10.9)	9.6	1.1 (0.3)	1.0 (1–2)	21.6**	1.3 (0.9)	1.0 (1–4)
MRI	31 (5.8)	5.2	1.0 (0.0)	1.0	10.9	1.3 (0.5)	1.0 (1–2)
TCD	20 (3.8)	2.9	1.2 (0.4)	1.0 (1–2)	10.8*	1.3 (0.5)	1.0 (1–2)
Electroencephalography	30 (5.7)	5.2	1.3 (0.6)	1.0 (1–3)	9.9	1.6 (0.9)	1.1 (1–3)
**Any tests**	**91 (17.3)**	**16.3**	N/A	N/A	**25.3**	N/A	N/A

*Abbreviations: N* Number, *EM* Episodic Migraine, *CM* Chronic Migraine, *SD* Standard Deviation, *TCM* Traditional Chinese Medicine, *CT* Computed Tomography, *MRI* Magnetic Resonance Imaging, *TCD* Transcranial Doppler ultrasonography, *N/A* Not Applicable

^a^Chi-square tests (or Fisher’s exact tests) were used to compare the use rates between EM and CM respondents, while the Mann–Whitney *U* tests were used to compare the number of resources used: no star, *p*-value > 0.05

^*^p-value < 0.05

^**^p-value < 0.01

**Table 2:** Acute medication utilisation in the preceding month and preventive medication utilisation in the preceding year

Medicine	All patients N (%)	EM (*N* = 466)						CM (*N* = 59)^b^					
		**%**		**Mean days used (SD)**		**Median (range)**		**%**		**Mean days used (SD)**			**Median (range)**
**Acute medicines**													
**Traditional Chinese patent medicines**^**a**^													
Gastrodia Capsule	21 (4.1)	4.4		7.2 (8.7)		5 (1–30)		1.0		15			15
Tou tongning Capsule	14 (2.7)	2.3		6.5 (9.1)		3.7 (1–30)		5.8		21.2 (15.0)			30 (3–30)
Yangxue Qingnao Granule	6 (1.1)	1.2		6 (6.4)		3.9 (1–18)		0		N/A			N/A
Zhengtian Pill	5 (0.9)	1.0		17 (11.3)		16.3 (3–30)		0		N/A			N/A
Tablet of Corydalistuber for Alleviating Pain	5 (0.9)	1.0		2.7 (1.6)		2.5 (1–5)		0		N/A			N/A
Seven Leaves Spirit Calmness Tablet	5 (0.9)	0.8		2		2		1.9		30			30
Lingyangjiao Pill	4 (0.8)	0.8		5 (3.5)		6 (1–8)		0		N/A			N/A
Tongtian Oral Liquid	4 (0.8)	0.8		7.6 (6)		10.4 (1–14)		0		N/A			N/A
Duliang Soft Capsule	2 (0.5)	0.5		3.3 (3.3)		3 (1–6)		0		N/A			N/A
999 Ganmao Ling Keli	2 (0.5)	0.4		1.6 (0.7)		1.7 (1–2)		0		N/A			N/A
Ershiwuwei Shanhu Wan	1 (0.2)	0.3		1		1		0		N/A			N/A
**Western medicines**													
Aspirin	127 (24.1)	23.8		4.5 (5.8)		2 (1–30)		26.7		8.1 (9.9)			3 (1–30)
Non-Aspirin NSAIDs	168 (32.0)	31.1		4.7 (5.8)		3 (1–30)		38.7		6.2 (7.2)			3 (1–30)
Acetaminophen (Paracetamol)	52 (9.9)	9.6		7.4 (9.4)		3.0 (1–30)		12.2		11.9 (11.8)			9.4 (1–30)
Triptans	10 (1.9)	1.1		7.5 (10)		3.9 (2–30)		3.7		5.0 (1.3)			5 (4–6)
Ergot alkaloids	4 (0.8)	0.9		4.7 (1.4)		5 (3–6)		0		N/A			N/A
Weak opioids/opioids	4 (0.8)	0.5		3.4 (2)		3.2 (2–5)		2		12			12
**Medicine**	**All patients N (%)**		**EM (*N* = 466)**						**CM (*N* = 59)**^**b**^				
			**%**		**Mean days used (SD)**		**Median (range)**		**%**		**Mean days used (SD)**	**Median (range)**	
Japan EVE QUICK Painkiller	2 (0.5)		0.4		2		2		0		N/A	N/A	
Barbiturates	1 (0.2)		0.3		2		2		0		N/A	N/A	
Antiemetics	1 (0.2)		0.3		9		9		0		N/A	N/A	
Glucocorticoids	1 (0.2)		0.3		8		8		0		N/A	N/A	
Mannitol injection	1 (0.2)		0.3		5		5		0		N/A	N/A	
**Any acute medicines**	**261 (49.7)**		**47.9**		**N/A**		**N/A**		**63.6**		**N/A**	**N/A**	
**Preventive medicines**													
Calcium antagonists	52 (9.9)		10.4		14.7 (40.6)		7 (7–360)		5.4		35.8 (69.9)	7.6 (7–154)	
β1-receptor antagonists	6 (1.2)		1.1		9.1 (6.4)		7 (7–25)		1.9		220	220	
Antiepileptics	20 (3.7)		4.1		14.8 (14.9)		8.1 (7–66)		1.0		78	78	
Vitamin B2	20 (3.8)		3.7		33 (88.6)		7 (7–360)		4.4		24.3 (24)	21.5 (7–45)	
Coenzyme Q10	5 (1.0)		0.6		21.5 (13.6)		27.5 (7–30)		3.9		9.6 (3.3)	9.7 (7–12)	
Candesartan Cilexetil	8 (1.5)		1.6		10.4 (6.8)		7 (7–30)		1.0		85	85	
Prednisone	4 (0.7)		0.5		13.3 (13.2)		7.9 (7–30)		2.0		36	36	
Duliang Soft Capsule	1 (0.2)		0.2		30		30		0		N/A	N/A	
Yangxue Qingnao Granule	1 (0.2)		0.2		30		30		0		N/A	N/A	
**Any preventive medicines**	**93 (17.8)**		**18.3**		**N/A**		**N/A**		**13.6**		**N/A**	**N/A**	
**Any medicines**	**276 (52.5)**		**51.0**		**N/A**		**N/A**		**64.6**		**N/A**	**N/A**	

*Abbreviations: N* Number, *EM* Episodic Migraine, *CM* Chronic Migraine, *SD* Standard Deviation, *NSAID* Non-Steroidal Anti-Inflammatory Drugs, *N/A* Not Applicable

^a^With the advancement of traditional Chinese medicine, Chinese herbal tonics have evolved into what are known as traditional Chinese patent medicines. These medicines are widely employed in clinical practice in China and are available in various forms like pills, capsules, or syrups

^b^The Chi-square tests (or Fisher’s exact tests) were used to compare the use rates between EM and CM respondents, while the Mann–Whitney *U* tests were used to compare the number of resources used: no star, *p*-value > 0.05

Notably, only 21.8% of the respondents reported attending an outpatient consultation. Among those who did not attend, 43.2% of the overall migraine positives (227 out of 525), did not utilise any other healthcare resources for their migraines either, including diagnostic tests, medicines, and complementary therapies.

Ninety-one respondents, constituting 17.3% of the sample, reported using at least one migraine-related diagnostic test, with Computed Tomography (CT) scan being the most commonly used test (57 out of 91 people). Furthermore, statistical analysis revealed significantly higher usage rates of CT scan (21.6% for CM, 9.6% for EM, *p* < 0.01) and Transcranial doppler ultrasonography (TCD) (10.8% for CM, 2.9% for EM, *p* < 0.05) among CM patients compared to EM patients.

Non-aspirin non-steroidal anti-inflammatory drugs (NSAIDs), including Ibuprofen, Naproxen, Diclofenac, and compounds of non-steroidal anti-inflammatory drugs, acetaminophen and caffeine, were the most commonly used acute medicines (32.0%). They were followed by Aspirin, which was used by 24.1% of the respondents. There was no significant difference in medicine utilisation between CM and EM respondents. Among those diagnosed with CM, a small proportion (13.6%) reported initiating preventive medicines, while the majority (63.6%) continued to take acute medicines when needed.

**Fig. 3:**
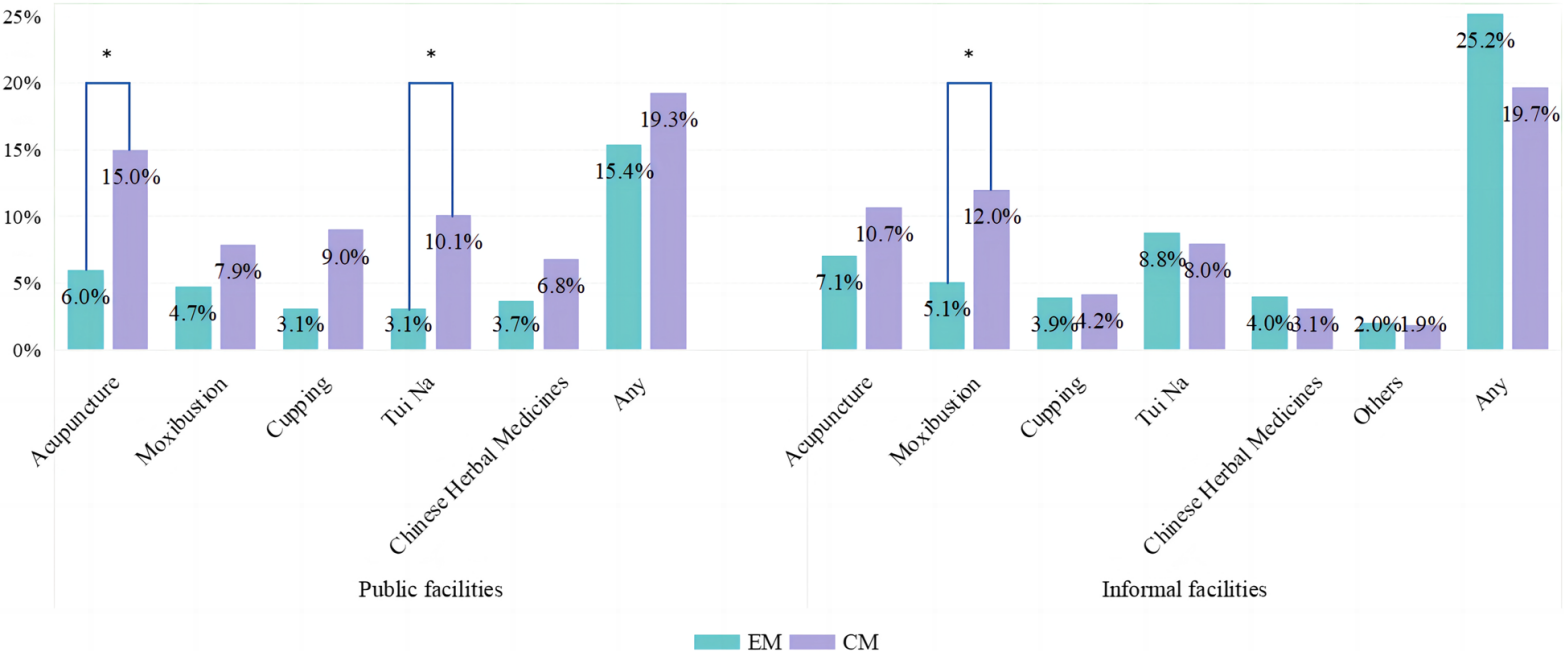
Complementary therapy utilisation in the preceding year Abbreviations: EM, Episodic Migraine; CM, Chronic Migraine. Note: Tui Na refers to Chinese massage therapy Chi-square tests/Fisher’s exact tests: no star, *p*-value > 0.05; *, *p*-value < 0.05

In total, 27.1% of migraine patients used complementary therapies, either at public or informal facilities. Figure 3 depicts the utilisation of complementary therapies among individuals with EM and CM. CM patients differed from EM patients in the usage rates of acupuncture (15.0% for CM, 6.0% for EM, *p* < 0.05) and Tui Na (Chinese massage therapy) (10.1% for CM, 3.1% for EM, *p* < 0.05) at public facilities. Additionally, CM patients differed from EM patients in the usage rates of moxibustion at informal facilities (12.0% for CM, 5.1% for EM, *p* < 0.05).

### Economic burden of migraines

**Fig. 4:**
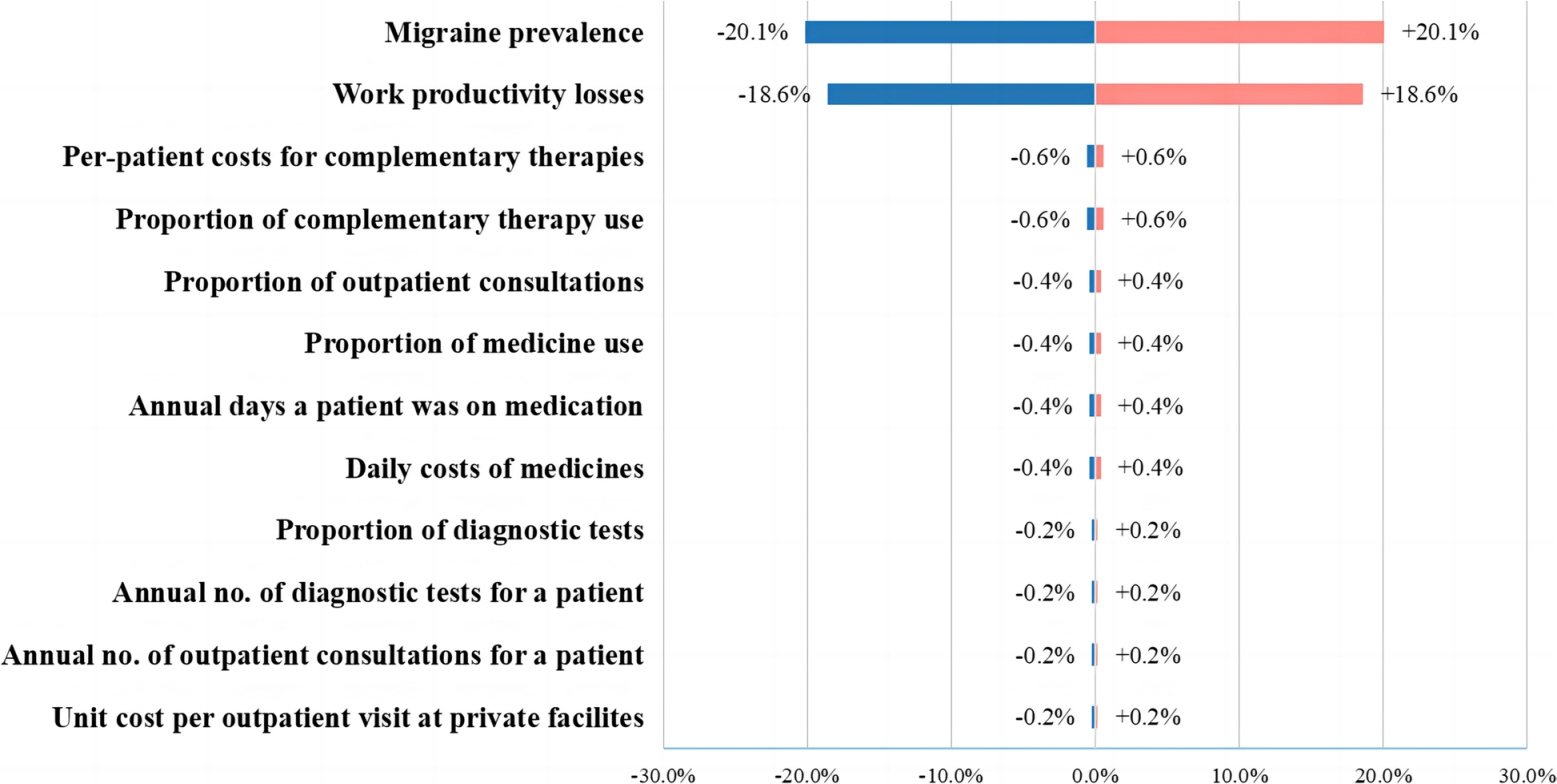
Tornado diagram showing the results of one-way sensitivity analysis Note: The ± percentages in the diagram indicate the changes in the total costs of migraines compared to the point estimate of $49,055.5 thousand (refer to Supplementary Material 4), based on a ± 20% change in each key parameter

Supplementary Material 4 provides the details of the point estimates of the annual costs of migraines. The impacts of a ± 20% change in each key parameter on the point estimate for the total costs are shown in Fig. 4. The prevalence of migraines and work productivity losses emerged as the predominant cost drivers.

**Fig. 5:**
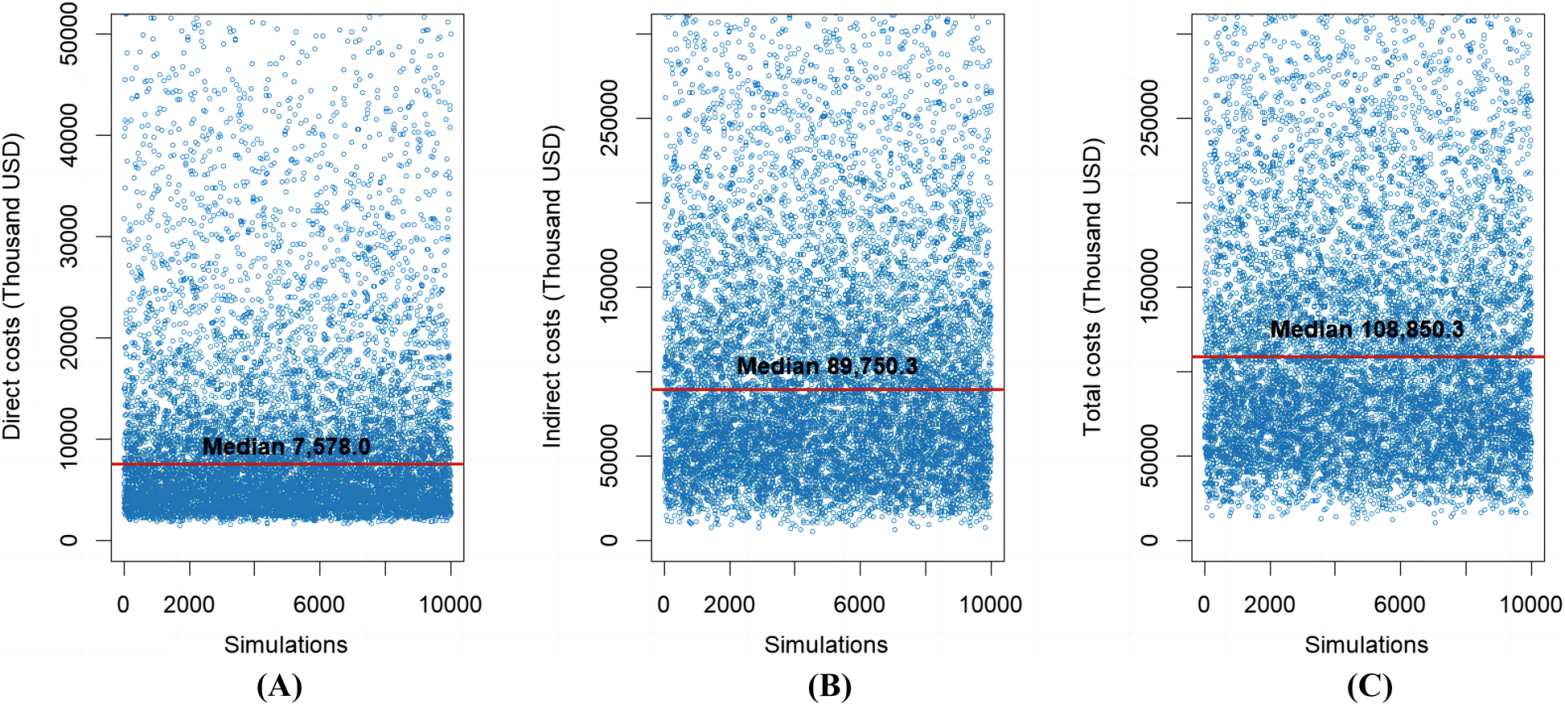
Annual costs from Monte Carlo simulations on the probabilistic model in 2022 USD: (**A**) Direct costs, (**B**) Indirect costs, and (**C**) Total costs

Figure 5 shows the results of the probabilistic sensitivity analyses, depicting the ranges of variations in the cost results. Due to the skewed distributions of the costs, medians along with 25th to 75th percentiles were reported. The Monte Carlo simulations on the probabilistic model produced the annual direct costs to the healthcare system of $7,578.0 thousand (25th to 75th percentile $4,509.2–$16,434.9 thousand), the annual indirect costs to employers of $89,750.3 thousand (25th to 75th percentile $53,211.6–$151,162.2 thousand), and the annual total costs to society of $108,850.3 thousand (25th to 75th percentile $67,370.1–$181,048.6 thousand). The analyses found that the majority of the total costs of migraines were borne by the employers, which in this study refer specifically to the banking sector. For the societal cost per patient-year, the model outputted a median value of $3,078.1.

## Discussion

### Findings of this study

To our best knowledge, this is the first population-based survey on migraine-related healthcare utilisation patterns among bank employees in China. In a representative sample of bank employees with migraines, only 21.8% reported seeking outpatient consultations, 52.5% reported taking medicines, and 27.1% reported using complementary therapies. Notably, nearly half of the migraine sufferers refrained from seeking medical care for their conditions. The observed low rates of healthcare utilisation among these migraine sufferers should not be interpreted as a diminished demand for healthcare. Rather, the observations point to a potential lack of access to appropriate care, as migraines are often persistent, which can significantly affect daily activities and overall well-being, underlining the importance of accessible and effective care.

The observed low rates of healthcare utilisation are consistent with similar studies conducted among bank employees in Malaysia [12] and the general Chinese population [26]. A recent review summarised the reasons for the inadequacy of migraine care, including social and clinical barriers, as well as the limited allocation of health resources to migraine care [37]. At the societal level, migraines are often not recognised as a serious health issue by the government, healthcare providers, employers, insurance agencies, and even patients themselves [38]. Instead, this condition is frequently downplayed as a minor ailment. Clinically, incorrect diagnosis and inappropriate management continue to be major problems. For example, in China, patients were reportedly often misdiagnosed with ‘nervous headache’, a non-existent disease [26]. This underscores deficiencies in healthcare provider training, which may explain why migraine sufferers lose trust in healthcare providers [39], thereby impeding their inclination to seek medical care. Moreover, the limited allocation of health resources to migraine care poses a global challenge. Many countries allocate their health budgets primarily to managing infectious diseases like tuberculosis and COVID-19, often overlooking the significant disability and economic burden associated with migraines.

It is also crucial to mention that stigma towards migraines, especially among patients experiencing a decrease in quality of life, may exhibit behavioural responses that influence their healthcare-seeking behaviours [40, 41]. Internalised stigma involves negative self-perceptions, resulting in feelings of shame, guilt, and isolation. This may lead individuals with migraines to question the legitimacy of their conditions and hesitate to seek assistance due to concerns over judgment or discrimination [42]. Externalised stigma encompasses discrimination and negative attitudes from healthcare providers, employers, and society, driven by misconceptions of migraines as merely headaches and a lack of awareness of their debilitating impacts [43]. These factors are essential considerations for future research to gain a comprehensive understanding of healthcare-seeking behaviours among migraine patients. Our findings underscore the urgent need to raise awareness and understanding of migraines among all stakeholders, and to optimise the organisation of the migraine care system. Such improvements are instrumental in enhancing health outcomes and providing better support for individuals affected by migraines.

The proportion of outpatient consultations observed in this study is lower than those in developed countries [10, 21], which may be attributed to the variations in economies and healthcare systems across countries. Of important note, the proportion of outpatient consultations in this study (21.8%) is approximately half of that observed in the broader population in China (52.9%) [26], despite a higher prevalence of migraines among bank employees than the general population, as reported in our earlier publication [11]. This discrepancy may likely be explained by two primary factors:

Firstly, it might be influenced by geographic inequality in healthcare resources and access across China’s diverse regions. In this country, wealthier provinces possess greater financial resources for healthcare services, with approximately 30% of provinces drawing 50% of high-quality healthcare resources [44]. Our survey was conducted in Guizhou province, which is characterised as economically disadvantaged and experiences a lower healthcare resource supply and utilisation compared to its more developed counterparts [45]. This underscores the necessity for targeted policy attention in disadvantaged provinces.

Secondly, the lower utilisation of outpatient care observed in this study may be attributed to the nature of the banking occupation. Previous research has identified a correlation between certain higher socio-economic status occupations and a decreased utilisation of outpatient care [46]. This trend is not solely shaped by socio-demographic factors and health status but is also influenced by patients’ knowledge and attitudes [46]. In China, the banking occupation undoubtedly holds a high socio-economic status, ranking third in average wage among all 19 occupations, as reported by the National Bureau of Statistics of China [47]. In the context of this study, the lower utilisation of outpatient care among bank employees may result from their knowledge and attitudes towards healthcare, fostering a belief in their ability to manage migraines independently [48]. Further investigation is imperative to comprehensively understand the factors influencing outpatient care utilisation among migraine patients. This will empower medical professionals to effectively provide the necessary diagnosis and care to individuals suffering from migraines.

The study also found that, apart from those seeking outpatient care, approximately 30% of migraine-positive respondents were self-medicating, mainly relying on acute medicines to alleviate symptoms. The most commonly used acute medicines were NSAIDs. Not only are NSAIDs extensively recommended in clinical practice guidelines, but they are also broadly utilised globally [1]. Furthermore, ample evidence exists supporting their cost-effectiveness in migraine management, including in low-income countries [1].

However, only a small 1.9% of migraine-positive respondents used triptans. This observation is corroborated by insights from expert interviews conducted during the content validation process for the HARDSHIP healthcare utilisation questionnaire (as detailed in Supplementary Material 1), highlighting the limited use of triptans. This is important given that triptans are recommended as an initial acute treatment option for migraines in the Chinese clinical practice guidelines [49], noted for their effectiveness and safety [2]. The under-utilisation can be attributed to the limited number of triptan categories available in the Chinese market (three categories, compared to seven in the U.S.) [27], their high prices, and the uncertainty regarding their reimbursement by health insurance [1]. For instance, sumatriptan, a branded medication, was priced at $101.5 for a two-unit package in 2022 USD, as per leading online retailers in China. Additionally, the low rates of seeking medical advice might contribute to individuals with migraines having a low awareness of better disease-specific treatments. These factors collectively likely deter migraine sufferers from obtaining and utilising triptans as their preferred treatment option.

Importantly, caution is advised concerning the frequent use of acute medicines. The clinical practice guidelines for migraine management in China [49] are consistent with those in Europe [50] and the U.S. [51], advocating sequencing and layering of acute and preventive treatments. Both NSAIDs and triptans, as discussed earlier, are acute medicines. The present study revealed that even among individuals experiencing headaches for 15 days or more per month, a worrying 63.6% persisted with inappropriate treatments by relying solely on acute medicines. Clinical practice guidelines recommend discontinuing acute treatments and considering the initiation of preventive treatments when an individual has more than two migraine attacks per month, encounters failed acute therapeutic attempts, or faces severe disruption in daily activities due to migraine [49, 52]. Failure to follow these recommendations increases the risks of the progression from EM to CM and the development of medication-overuse headaches, which can further complicate conditions and present enormous challenges for clinical management [53]. Preventive treatments that are highly recommended by the Chinese clinical practice guidelines include flunarizine, topiramate, valproate, beta blockers, tricyclic antidepressants, and venlafaxine [49]. However, it should be noted that many of these preventive treatments are limited by moderate efficacy, moderate to high rates of adverse events, contraindications, or interactions that limit use [51]. Accordingly, monitoring patients’ responses to treatment is essential.

The issue of medicine misuse is not unique to the present study; it is a widespread concern globally. Even in Western countries with well-developed migraine care systems, abundant financial resources, and availability of specialist care, less than one-third of individuals with higher migraine frequency reported using preventive treatments [10]. An implication of these findings is the critical importance of seeking medical consultations rather than resorting to self-medication, especially for individuals with severe migraine symptoms and disabilities. Educating patients about the negative consequences of excessive use of acute medicines and the adoption of appropriate migraine management strategies is crucial.

Another notable finding to highlight is that, to the best of our knowledge, this study is the first investigation into the patterns of complementary therapy usage among migraine sufferers in China. These usage patterns observed in this study, either at public or informal facilities, are consistent with observations made in other countries, indicating that nearly a third of patients had utilised complementary therapies for migraines in the past year [54, 55]. A remarkable discovery was that one study found that 84% of migraine patients had utilised complementary therapies in their lifetime [55]. Currently, the evidence on the efficacy of these complementary therapies is limited but promising. A meta-analysis found that acupuncture therapies demonstrated greater efficacy for migraines and elicited fewer adverse reactions compared to conventional drug therapies [56]. Another meta-analysis revealed a statistically significant difference (risk ratio = 1.25, 95%CI 1.19–1.32) in efficacy rates of migraine management between Tui Na therapy and conventional drug therapies, with Tui Na therapy demonstrating superiority [57]. Despite these findings, the quality of the evidence raises concerns, as certain trials are susceptible to biases in their design and implementation [56, 57]. Therefore, while these results are encouraging, more high-quality studies on complementary therapies for migraines are needed in the future.

In alignment with previous research [10, 58], this study underscores a significant disparity in healthcare utilisation related to migraines between CM and EM patients. Specifically, this study revealed that CM patients were 3.1 times more likely to seek outpatient consultations at tertiary-level hospitals, where specialised care is more accessible. Also, CM patients were 2.3 and 3.7 times more likely to undergo CT and TCD tests, respectively, and were two to four times more likely to opt for various types of complementary therapies compared to EM patients. Considering the increased healthcare utilisation associated with CM, this study illuminates an urgency of preventing the progression from EM to CM. Effectively prevention of this progression can significantly reduce the overall healthcare burden attributed to migraines.

This study represents the most recent and comprehensive estimation of the economic burden of migraines in China thus far. It also uniquely employs a micro-costing approach, which entails assigning unit costs to various aspects of migraine-related healthcare utilisation. This costing approach is considered to be more comprehensive and accurate compared to alternative approaches, and it has been recognised as the preferred approach for COI studies [22, 30]. Moreover, our utilisation of primary data collected from a representative sample enhances the generalisability of the economic burden estimates. Furthermore, the robustness of the cost estimation is further strengthened through the use of a probabilistic analysis, resulting in a median societal cost per migraine patient of $3,078.1 in 2022 USD. The cost estimates in this study serve as an evidence-based benchmark for comparison. This benchmark is instrumental for assessing the potential economic savings from developing and implementing programmes aimed at improving migraine management for the banking sector in Guizhou province. Additionally, these cost estimates establish a foundational measurement for future health economic evaluations. They provide a baseline against which the effects of various policies, services, or interventions can be assessed.

The societal cost per patient-year for migraine in this study, at $3,078.1, exceeds the costs associated with several other chronic diseases that have attracted attention from researchers and policymakers in China. For instance, in 2022 USD, the per-patient-year societal cost is $2,206.6 for type 2 diabetes [59] and $2,113.8 for chronic hepatitis B [60]. These comparisons offer evidence-based insights for policymakers, aiding them in establishing priorities in policy formulation and health intervention investments. While the present study focused on the banking employees in Guizhou province, unlike other referenced studies that covered the general population, its findings shed light on the burden of migraines within this specific occupational population. This is pivotal in drawing the attention of Chinese policymakers to prioritise migraine policies, extending beyond the banking sector to potentially include other office-based occupations. This consideration holds particular relevance, given the similarities in environments and exposures between banks and other office settings, highlighting a broader generalisability of the findings for occupational health management across various sectors.

As previously discussed, nearly half of the migraine sufferers reported not seeking any medical care. Although their migraine episodes do not present to the healthcare system, these episodes still pose a substantial burden on employers and society. Our findings align with previously published research [61, 62], indicating that the indirect costs account for the vast majority of the economic burden of migraines. The relative proportion of indirect costs in the total costs varies across different diseases. In conditions like migraine, indirect costs constitute a major component of the total costs, while in conditions like cancer, direct medical costs are predominant [63]. A four-year cohort study found that migraines significantly reduced work productivity for employees and emerged as one of the costliest conditions for employers [64]. This is a vital lesson for organisations to invest in migraine prevention and control. Such investments are not only beneficial for employee well-being but are also expected to yield a favourable return.

### Limitations

Some limitations of this study require consideration. The observed healthcare utilisation patterns and estimated costs are specific to bank employees in Guizhou province, China. As highlighted in our previous study [11], there is a notably higher prevalence of migraines among bank employees in Guizhou province compared to the general population in China. Therefore, it is acknowledged that there are potential limitations in the external validity of the findings for the entire country, given the absence of a more representative and diverse sample. Nonetheless, it is worth emphasising that this study managed to obtain a representative sample of employees from the banking sector in Guizhou. This enables the findings to be generalised to the bank employee population within Guizhou, providing informative insights for this specific province. Moreover, these insights could have wider implications for other workers in office-based occupations, particularly considering the similarities in environments and exposures between banks and other office settings.

The next limitation pertains to the structure of the model. All models, by their nature, are abstractions of reality and cannot fully take into account every clinical pathway. In this study, despite the validation of the model, certain cost components such as direct non-medical costs, were not included in the model. However, this exclusion is based on the justification that direct non-medical costs tend to be minimal for migraine sufferers, as individuals with headache disorders seldom require social services or special transportation for health-seeking [20]. Also, this study did not assess indirect costs associated with lifestyle compromises, the burden imposed on caregivers, and potential career repercussions resulting from migraines. However, it is important to recognise that estimating the economic consequences of these events is a complex undertaking, as indicated by a 2021 review [1]. Similarly, this study did not include intangible costs, such as reduced quality of life and stigma, as cost components due to challenges in measurement and ongoing controversies over their inclusion in COI studies [65–67]. It is important to highlight that these aspects have financial impacts as well. Although the willingness-to-pay approach has been argued to be able to cover all the financial impacts of a disease, the WHO notes that the estimates derived from such an approach are susceptible to considerable uncertainty [33]. In fact, guidelines recommend that models should not be overly complex than necessary to capture all pertinent aspects of the system being modelled [68]. Maintaining simplicity in model structure ensures that a model remains both practical and useful.

Thirdly, in this study, the reliance on self-diagnosis of migraines, as well as self-reported healthcare utilisation and productivity losses, could potentially introduce biases, such as inaccurate recall and false reporting. Despite these potential limitations, the survey data represent the best available source of information for the economic analysis, particularly in light of this study’s specific focus on the targeted population. Moreover, the sensitivity analyses conducted were pivotal in adjusting the results to account for potential biases.

Finally, the estimation of medicine costs in this study is conservative. This can be attributed to two main aspects. One such aspect is the use of daily doses recommended on the medicine labels, which might not fully capture individual-specific variations in medicine usage. While the recommended daily doses offer general guidance, they may not accurately reflect the unique needs and variations among migraine patients with different conditions. Nevertheless, estimating the average daily costs for medicines based on recommended daily doses is a practical approach for cost estimation and helps minimise recall bias. The other aspect is the adoption of the cheapest prices for OTC medicines. In this study, it was assumed that all migraine patients prefer the cheapest medicines, which may not accurately reflect the preferences and choices of every patient. However, the decision was based on the rational choice theory in economics, suggesting that consumers often seek to maximise their utility while minimising costs [69]. Despite the above-mentioned limitations, this study holds significant value and should serve as a catalyst for Chinese policymakers to recognise migraines as a substantial burden on society, impacting the healthcare system, workplace productivity, and individual well-being.

## Conclusions

This study highlights concerning trends in migraine management among bank employees in China. Nearly half of the respondents with migraines did not seek medical care, and among those experiencing frequent migraines, a significant portion were using inappropriate treatments, potentially worsening their conditions. The healthcare utilisation patterns revealed by this study underscore the urgent need to improve awareness and understanding of migraines across all stakeholders. Additionally, there is a pressing necessity to improve the organisation of the migraine care system, and to educate patients about the detrimental effects of excessive acute medication use and the importance of adopting appropriate migraine management strategies.

Migraines impose a significant economic burden on the healthcare system, employers, and society at large. The cost estimates provided in this study offer evidence-based benchmarks for assessing potential economic savings from implementing programmes to enhance migraine management in the banking sector. These findings are crucial for urging Chinese policymakers to prioritise migraine policies, extending beyond the banking sector to other office-based occupations.

### Supplementary Information

**Additional file 1: Supplementary Material 1.** Adaptation and validation of the HARDSHIP healthcare utilisation questionnaire.

**Additional file 2: Supplementary Material 2.** Unit costs of healthcare resources in China (in 2022 USD).

**Additional file 3: Supplementary Material 3.** Model inputs and data sources for estimating the economic burden of migraines among bank employees.

**Additional file 4: Supplementary Material 4.** Point estimation of the economic burden of migraines among bank employees (in 2022 USD).

## Data Availability

The datasets generated and/or analysed during this study are available upon reasonable request from the corresponding authors.

## Abbreviations

EM: Episodic Migraine
CM: Chronic Migraine
U.S.: United States
USD: United States dollars
GDP: Gross Domestic Product
WHO: World Health Organization
SD: Standard Deviation
COI: Cost-Of-Illness
CT: Computed Tomography
TCD: Transcranial Doppler ultrasonography
NSAIDs: Non-Steroidal Anti-Inflammatory Drugs
TCM: Traditional Chinese Medicine
MRI: Magnetic Resonance Imaging
Tui Na: Chinese massage therapy

## References

[1] Ashina M, Katsarava Z, Do TP, Buse DC, Pozo-Rosich P, Özge A (2021). Migraine: epidemiology and systems of care. Lancet.

[2] Ashina M, Buse DC, Ashina H, Pozo-Rosich P, Peres MFP, Lee MJ (2021). Migraine: integrated approaches to clinical management and emerging treatments. Lancet.

[3] Ashina M, Terwindt GM, Al-Karagholi MA, de Boer I, Lee MJ, Hay DL (2021). Migraine: disease characterisation, biomarkers, and precision medicine. Lancet.

[4] Steiner TJ, Terwindt GM, Katsarava Z, Pozo-Rosich P, Gantenbein AR, Roche SL (2022). Migraine-attributed burden, impact and disability, and migraine-impacted quality of life: expert consensus on definitions from a Delphi process. Cephalalgia.

[5] GBD (2019). Diseases and Injuries Collaborators (2020) Global burden of 369 diseases and injuries in 204 countries and territories, 1990–2019: a systematic analysis for the Global Burden of Disease Study 2019. Lancet.

[6] Kurth T, Winter AC, Eliassen AH, Dushkes R, Mukamal KJ, Rimm EB (2016). Migraine and risk of cardiovascular disease in women: prospective cohort study. BMJ.

[7] Øie LR, Kurth T, Gulati S, Dodick DW (2020). Migraine and risk of stroke. J Neurol Neurosurg Psychiatry.

[8] Katsarava Z, Mania M, Lampl C, Herberhold J, Steiner TJ (2018). Poor medical care for people with migraine in Europe - evidence from the Eurolight study. J Headache Pain.

[9] Takeshima T, Wan Q, Zhang Y, Komori M, Stretton S, Rajan N (2019). Prevalence, burden, and clinical management of migraine in China, Japan, and South Korea: a comprehensive review of the literature. J Headache Pain.

[10] Bloudek LM, Stokes M, Buse DC, Wilcox TK, Lipton RB, Goadsby PJ (2012). Cost of healthcare for patients with migraine in five European countries: results from the International Burden of Migraine Study (IBMS). J Headache Pain.

[11] Wei D, Loganathan T, Wong LP (2023). Employees of the banking sector in Guizhou Province in China: prevalence of migraine, symptoms, disability and occupational risk factors. J Headache Pain.

[12] Wong LP, Alias H, Bhoo-Pathy N, Chung I, Chong YC, Kalra S (2020). Impact of migraine on workplace productivity and monetary loss: a study of employees in banking sector in Malaysia. J Headache Pain.

[13] Burton WN, Conti DJ, Chen C-Y, Schultz AB, Edington DW (2002). The economic burden of lost productivity due to migraine headache: a specific worksite analysis. J Occup Environ Med.

[14] Friedman DI, De ver Dye T (2009). Migraine and the environment. Headache.

[15] Barber M, Pace A (2020). Exercise and migraine prevention: a review of the literature. Curr Pain Headache Rep.

[16] Ahn AH (2013). Why does increased exercise decrease migraine?. Curr Pain Headache Rep.

[17] LaBan MM, Meerschaert JR (1989). Computer-generated headache. Brachiocephalgia at first byte. Am J Phys Med Rehabil.

[18] Peroutka SJ (2014). What turns on a migraine? A systematic review of migraine precipitating factors. Curr Pain Headache Rep.

[19] Borsook D, Maleki N, Becerra L, McEwen B (2012). Understanding migraine through the lens of maladaptive stress responses: a model disease of allostatic load. Neuron.

[20] Linde M, Gustavsson A, Stovner LJ, Steiner TJ, Barré J, Katsarava Z (2012). The cost of headache disorders in Europe: the Eurolight project. Eur J Neurol.

[21] Bonafede M, Sapra S, Shah N, Tepper S, Cappell K, Desai P (2018). Direct and indirect healthcare resource utilization and costs among migraine patients in the United States. Headache.

[22] Stovner LJ, Al Jumah M, Birbeck GL, Gururaj G, Jensen R, Katsarava Z (2014). The methodology of population surveys of headache prevalence, burden and cost: principles and recommendations from the Global Campaign against Headache. J Headache Pain.

[23] Wei D, Wong LP, Loganathan T, Tang R-R, Chang Y, Zhou H-N (2022). Validation studies on migraine diagnostic tools for use in nonclinical settings: a systematic review. Arq Neuropsiquiatr.

[24] Yu S-Y, Cao X-T, Zhao G, Yang X-S, Qiao X-Y, Fang Y-N (2011). The burden of headache in China: validation of diagnostic questionnaire for a population-based survey. J Headache Pain.

[25] Li X, Zhou J, Tan G, Wang Y, Ran L, Chen L (2012). Diagnosis and treatment status of migraine: a clinic-based study in China. J Neurol Sci.

[26] Liu R, Yu S, He M, Zhao G, Yang X, Qiao X (2013). Health-care utilization for primary headache disorders in China: a population-based door-to-door survey. J Headache Pain.

[27] Yu S, Zhang Y, Yao Y, Cao H (2020). Migraine treatment and healthcare costs: retrospective analysis of the China Health Insurance Research Association (CHIRA) database. J Headache Pain.

[28] Luo N, Qi W, Zhuang C, Di W, Lu Y, Huang Z (2014). A satisfaction survey of current medicines used for migraine therapy in China: Is Chinese patent medicine effective compared with Western medicine for the acute treatment of migraine?. Pain Med.

[29] Husereau D, Drummond M, Augustovski F, de Bekker-Grob E, Briggs AH, Carswell C (2022). Consolidated health economic evaluation reporting standards 2022 (CHEERS 2022) statement: updated reporting guidance for health economic evaluations. Int J Technol Assess Health Care.

[30] Xu X, Lazar CM, Ruger JP (2021). Micro-costing in health and medicine: a critical appraisal. Health Econ Rev.

[31] Steiner TJ, Gururaj G, Andrée C, Katsarava Z, Ayzenberg I, Yu S-Y (2014). Diagnosis, prevalence estimation and burden measurement in population surveys of headache: presenting the HARDSHIP questionnaire. J Headache Pain.

[32] Eddy DM, Hollingworth W, Caro JJ, Tsevat J, McDonald KM, Wong JB (2012). Model transparency and validation: a report of the ISPOR-SMDM Modeling good research practices task force-7. Value Health.

[33] World Health Organization (2009). WHO guide to identifying the economic consequences of disease and injury.

[34] National Health Commission of the People’s Republic of China (2023) Statistical Communiqué of the People's Republic of China on the 2022 National Healthcare Development. http://www.nhc.gov.cn/guihuaxxs/s3585u/202309/6707c48f2a2b420fbfb739c393fcca92/files/9b3fddc4703d4c9d9ad399bcca089f03.pdf. Accessed April 2024.

[35] Drummond MF, Sculpher MJ, Claxton K, Stoddart GL, Torrance GW (2015). Methods for the economic evaluation of health care programmes.

[36] Guizhou Provincial Bureau of Statistics (2023). 2022 Guizhou Statistical Yearbook.

[37] Atif M, Hussain S, Sarwar MR, Saqib A (2017). A review indicating the migraine headache as a prevalent neurological disorder: still under-estimated, under-recognized, under-diagnosed and under-treated. J Pharm Pract Community Med.

[38] Bigal M, Krymchantowski AV, Lipton RB (2009). Barriers to satisfactory migraine outcomes. What have we learned, where do we stand?. Headache.

[39] Katić BJ, Krause SJ, Tepper SJ, Hu HX, Bigal ME (2010). Adherence to acute migraine medication: What does it mean, why does it matter?. Headache.

[40] Raggi A, Giovannetti AM, Quintas R, D'Amico D, Cieza A, Sabariego C (2012). A systematic review of the psychosocial difficulties relevant to patients with migraine. J Headache Pain.

[41] Estave PM, Beeghly S, Anderson R, Margol C, Shakir M, George G (2021). Learning the full impact of migraine through patient voices: a qualitative study. Headache.

[42] Parikh SK, Kempner J, Young WB (2021). Stigma and migraine: developing effective interventions. Curr Pain Headache Rep.

[43] Tana C, Raffaelli B, Souza MNP, de la Torre ER, Massi DG, Kisani N (2024). Health equity, care access and quality in headache - part 1. J Headache Pain.

[44] Zhang X, Zhao L, Cui Z, Wang Y (2015). Study on equity and efficiency of health resources and services based on key indicators in China. PLoS ONE.

[45] Zhang T, Xu Y, Ren J, Sun L, Liu C (2017). Inequality in the distribution of health resources and health services in China: hospitals versus primary care institutions. Int J Equity Health.

[46] Rinne H, Laaksonen M, Blomgren J (2022). Use of outpatient and inpatient health care services by occupation—a register study of employees in Oulu. Finland BMC Health Serv Res.

[47] National Bureau of Statistics of China (2022). China Population and Employment Statistics Yearbook.

[48] Bennadi D (2013). Self-medication: a current challenge. J Basic Clin Pharm.

[49] Chinese Neurologists Association (2022) 中国偏头痛诊治指南 (2022版). [Guidelines for the diagnosis and treatment of migraine in China (2022 Edition)]. Chinese Journal of Pain Medicine 28(12): 881–898. Retrieved from https://kns.cnki.net/kcms2/article/abstract?v=6xaVI2TORM3eFtmmRNdT4iXgrnTEdioULPsK7k2Vgq-27rrt1GwrrA3SLnBO1ba-T-mIGxZc0n9rLodAIS_c_fp-yH04ERa9yylhL2zEQ9euT9OR2a93_o0FmIqld6PMpXSr_ViB2odZmveLFN9Bng==&uniplatform=NZKPT&language=CHS

[50] Evers S, Áfra J, Frese A, Goadsby PJ, Linde M, May A (2009). EFNS guideline on the drug treatment of migraine – revised report of an EFNS task force. Eur J Neurol.

[51] Ailani J, Burch RC, Robbins MS, the Board of Directors of the American Headache Society (2021). The American Headache Society Consensus Statement: update on integrating new migraine treatments into clinical practice. Headache.

[52] Steiner TJ, Jensen R, Katsarava Z, Linde M, MacGregor EA, Osipova V (2019). Aids to management of headache disorders in primary care (2nd edition). J Headache Pain.

[53] Eigenbrodt AK, Ashina H, Khan S, Diener H-C, Mitsikostas DD, Sinclair AJ (2021). Diagnosis and management of migraine in ten steps. Nat Rev Neurol.

[54] Zhang Y, Dennis JA, Leach MJ, Bishop FL, Cramer H, Chung VCH (2017). Complementary and alternative medicine use among US adults with headache or migraine: results from the 2012 National Health Interview Survey. Headache.

[55] Adams J, Barbery G, Lui C-W (2013). Complementary and alternative medicine use for headache and migraine: a critical review of the literature. Headache.

[56] Ou M-Q, Fan W-H, Sun F-R, Jie W-X, Lin M-J, Cai Y-J (2020). A systematic review and meta-analysis of the therapeutic effect of acupuncture on migraine. Front Neurol.

[57] Tang X-Y, Shang W-R, Weng Z-W, Qiu R-J, Tian J-H, L, Y.-G. (2016) Massage for migraine: a meta-analysis. Traditional Medicine Research 1(1): 32–42. Retrieved from https://www.tmrjournals.com/public/articlePDF/20201130/48f13aba6eb3bd4c05e89e161ec02ae8.pdf

[58] Messali A, Sanderson JC, Blumenfeld AM, Goadsby PJ, Buse DC, Varon SF (2016). Direct and indirect costs of chronic and episodic migraine in the United States: a web-based survey. Headache.

[59] Wang W, McGreevey WP, Fu C, Zhan S, Luan R, Chen W, et al (2009) Type 2 diabetes mellitus in China: a preventable economic burden. Am J Manag Care 15(9): 593–601. Retrieved from https://www.ajmc.com/view/ajmc_09sep_wang_593to601 19747024

[60] Li X, Xu X, Li J, Huang Y, Wang C, Zhang Y (2022). Direct and indirect costs of allergic and non-allergic rhinitis to adults in Beijing. China Clin Transl Allergy.

[61] Hjalte F, Olofsson S, Persson U, Linde M (2019). Burden and costs of migraine in a Swedish defined patient population – a questionnaire-based study. J Headache Pain.

[62] Lublóy Á (2019). Economic burden of migraine in Latvia and Lithuania: direct and indirect costs. BMC Public Health.

[63] Schultz AB, Chen C-Y, Edington DW (2009). The cost and impact of health conditions on presenteeism to employers. Pharmacoeconomics.

[64] Allen D, Hines EW, Pazdernik V, Konecny LT, Breitenbach E (2018). Four-year review of presenteeism data among employees of a large United States health care system: a retrospective prevalence study. Hum Resour Health.

[65] Rice DP (2000). Cost of illness studies: What is good about them?. Inj Prev.

[66] Jo C (2014). Cost-of-illness studies: concepts, scopes, and methods. Clin Mol Hepatol.

[67] Larg A, Moss JR (2011). Cost-of-illness studies: a guide to critical evaluation. Pharmacoeconomics.

[68] Dahabreh IJ, Trikalinos TA, Balk EM, Wong JB (2016). Recommendations for the conduct and reporting of modeling and simulation studies in health technology assessment. Ann Intern Med.

[69] Lovett F (2006). Rational choice theory and explanation. Ration Soc.

