# Supplementary material for: Healthcare utilisation and economic burden of migraines among bank employees in China: a probabilistic modelling study
Source: J Headache Pain. 2024 Apr 19;25(1):60. doi: 10.1186/s10194-024-01763-w (PMC11027248; doi:10.1186/s10194-024-01763-w)
Supplement: Supplementary file 1 — Additional file 1: Supplementary Material 1. Adaptation and validation of the HARDSHIP healthcare utilisation questionnaire. [file 10194_2024_1763_MOESM1_ESM.docx]

**Adaptation and validation of the HARDSHIP healthcare utilisation questionnaire**

1. **Adaptation of the HARDSHIP healthcare utilisation questionnaire**

China’s healthcare service delivery system includes both public and private healthcare facilities [1], allowing patients the flexibility to seek healthcare services from various levels of care [2]. Public facilities are the predominant providers of healthcare services, and private facilities are important supplements to the healthcare delivery system [1]. China’s public healthcare system comprises clinics, primary-level hospitals, secondary-level hospitals, tertiary-level hospitals and Traditional Chinese Medicine (TCM) hospitals. Therefore, to align with the healthcare system in China, the response options related to outpatient consultations in the HARDSHIP healthcare utilisation questionnaire were modified to reflect these healthcare facilities commonly available in this country, including public clinics, public primary-level hospitals, public secondary-level hospitals, public tertiary-level hospitals, public TCM hospitals, and private facilities.

Furthermore, the questionnaire listed the diagnostic tests commonly used among Chinese migraine patients, including Computed Tomography (CT) scan, Magnetic Resonance Imaging (MRI), Transcranial Doppler ultrasonography (TCD), and electroencephalography [2-4], as identified through our review.

Next, the response options for migraine medicines were revised to reflect the use patterns of medicines among Chinese migraine patients, as documented in prior studies [3-5]. The clinical practice guidelines for migraine management in China [6] are consistent with those in Europe [7] and the U.S. [8], advocating sequencing and layering of acute and preventive treatments. Moreover, China has established guidelines that integrate traditional Chinese and Western medicine for the prevention and treatment of migraines [9]. The revisions made to the response options in the questionnaire involved updating the list of Western medicines for acute medication and incorporating commonly used traditional Chinese patent medicines^[[1]](#footnote-1)^. Although triptans are highly recommended as migraine-specific acute medicines in the clinical practice guidelines [10], only three types of triptans were included in the questionnaire, sumatriptan, zolmitriptan, and rizatriptan, due to their availability in China [4]. In addition, a list of migraine preventive medicines commonly used in China was provided [3-5] .

Finally, common complementary therapies for migraines include acupuncture, herbs, Tui Na (Chinese massage therapy), cupping, and moxibustion [11]. As such, questions and response options related to complementary therapies were added to the pre-final version of the HARDSHIP healthcare utilisation questionnaire.

**Table 1** Pre-final version of the HARDSHIP healthcare utilisation questionnaire

| **No** | **Question** | **Answer** |
| --- | --- | --- |
| 1 | Please look at these lists. Which of these have you used in the last month (no matter prescription or OTC)?  [enter by each medication the number of days on which you used it in the last month.] | □ Nothing at all  **Traditional Chinese patent medicines ^a^:**  □ Gastrodia Capsule  □ Zhengtian Pill  □ Lingyangjiao Pill  □ Duliang Soft Capsule  □ Yangxue Qingnao Granule  □ Ershiwuwei Shanhu Wan  □ Tongtian Oral Liquid  □ Tablet of Corydalistuber for Alleviating Pain  □ Seven Leaves Spirit Calmness Tablet  □ Tou Tongning Capsule  **Western medicines:**  □ Aspirin  □ Acetaminophen (Paracetamol)  □ Ibuprofen  □ Caffeine  □ Paracetamol, aminophenazone, caffeine, and chlorphenamine maleate tablets  □ Weak opioids/opioids  □ Ergot alkaloids  □ Antiemetics  □ Sumatriptan  □ Zolmitriptan  □ Rizatriptan  Number of days ________ |
| 2 | Are there any other medications you have used to treat your headaches in the last month? | Name of medications ________  Number of days ________ |

**Table 1** continued

| **No** | **Question** | **Answer** |
| --- | --- | --- |
| 3 | Medications to prevent headaches are usually taken daily. Are you taking any of these now? | □Sibelium (Flunarizine)  □ β1-receptor antagonists  □ Antiepileptics  □ Antidepressants  □ Type A botulinum toxin  □ Others ________(name)  ________how long taken |
| 4 | Have you had formal professional advice about your headaches in the last year? Who from, and how many times? | □ No one  □ Public clinics  □ Public primary-level hospitals  □ Public secondary-level hospitals  □ Public tertiary-level hospitals  □ Public TCM hospitals  □ Private facilities  Number of times ________ |
| 5 | Because of your headaches, have you had any of these tests in the last year? | □ CT scan  □ MRI  □ TCD  □ Electroencephalography  □ Others________(name) |
| 6 | Have you, in the last year, tried any complementary therapies at **public facilities**? | □ Acupuncture ________ (expenses)  □ moxibustion ________ (expenses)  □ cupping _____(expenses)  □ Tui Na_____(expenses)  □ Herbology_____(expenses)  □ Others______(name) _____(expenses) |
| 7 | Have you, in the last year, tried any complementary therapies at **private facilities**? | □ Acupuncture  □ Herbology  □ Tui Na  □ Cupping  □ Moxibustion  □ Others________(name)  Expense/year ________ |
| Abbreviations: OTC, Over-the-counter; TCM, Traditional Chinese Medicine; CT, Computed Tomography; MRI, Magnetic Resonance Imaging; TCD, Transcranial Doppler ultrasonography.  Note: Tui Na refers to Chinese massage therapy.  ^a^ With the advancement of TCM, Chinese herbal tonics have evolved into what are known as traditional Chinese patent medicines. These medicines are widely employed in clinical practice in China and are available in various forms like pills, capsules, or syrups. | | |

1. **Validation of the HARDSHIP healthcare utilisation questionnaire**

## 2.1 Methods of the questionnaire validation

The validation process primarily focused on content and face validity to ensure the questionnaire’s relevance, comprehensiveness, and comprehensibility in the target population. Since the response options in the HARDSHIP healthcare utilisation questionnaire only captured respondents’ health resource choices without providing measurable data, conducting cross-cultural validation for other psychometric properties such as structural or criterion validity was not feasible.

Content validity is defined as the extent to which the items in the questionnaire are thoroughly representative of the research domain [12]. In this research, six experts (three neurologists and three public health specialists) with more than eight years of experience were interviewed. They were asked to provide suggestions on how to improve the instructions and all items of the pre-final questionnaire. After reaching an 80% agreement among them, each expert was asked to evaluate the conceptual relevance, comprehensiveness, and comprehensibility of the items from the perspective of respondents, using a 4-point Likert scale, ranging from “inadequate” (score 1) to “very good” (score 4). Subsequently, item-level (I-CVI) and scale-level (S-CVI) content validity indexes were calculated to measure expert agreement [13]. The I-CVI is calculated as the proportion of experts scoring 3 or 4 among all experts. For the calculation of the S-CVI, there are two common methods, universal agreement (UA) and average (AVE) [13]. S-CVI_UA_ is calculated by dividing the number of items that receive an I-CVI of one by the total number of items in the questionnaire, while S-CVI_AVE_ is the average of the I-CVIs for all items in the questionnaire. When the I-CVIs, S-CVI_UA_ and S-CVI_AVE_ are above 0.78, 0.80, and 0.90, respectively, content validity is considered good [13].

Face validity is an assessment of the comprehensibility of the target population, taking into account their health literacy. A pilot sample size of 10 to 40 is recommended to ensure face validity [14]. Therefore, given the prevalence rate of migraines, a voluntary sample of 94 bank employees was invited to respond to the HARDSHIP diagnosis questionnaire. Of these, 38 participants with a positive diagnosis of migraine were asked to rate the clarity and format of the instructions and items in the pre-final version of the HARDSHIP healthcare utilisation questionnaire using a dichotomous scale (clear or unclear). If “unclear”, they were asked to provide suggestions for better presentation. Following that, these suggestions were discussed by the previously mentioned expert panel until a consensus was achieved, and a final version of the HARDSHIP healthcare utilisation questionnaire was produced.

- 1. **Results of the questionnaire validation**

Table **2** summarises the characteristics of the six experts involved in the content validation process. They ranged in age from 35 to 42 years old, with three males and three females. Three experts were clinical professionals in neurology, while the other three were public health specialists. They all had more than eight years of work experience.

**Table 2** Socio-demographics of the experts performing content validation

| **No** | **Age (years)** | **Gender** | **Majority** | **Education** | **Title** | **Work experience** |
| --- | --- | --- | --- | --- | --- | --- |
| 1 | 36 | Male | Clinical, Neurology | Postgraduate | Associate professor | 8 |
| 2 | 37 | Female | Clinical, Neurology | Ph.D. | Associate professor | 10 |
| 3 | 41 | Male | Clinical, Neurology | Postgraduate | Professor | 17 |
| 4 | 42 | Female | Academic, Public health | Ph.D. | Professor | 20 |
| 5 | 42 | Male | Academic, Public health | Postgraduate | Professor | 15 |
| 6 | 35 | Female | Academic, Public health | Ph.D. | Associate professor | 8 |

Abbreviation: Ph.D., Doctor of Philosophy.

According to the three neurologists, triptans are not widely available in most hospitals in China, limiting doctors from prescribing them as a treatment option for migraine patients. After discussion, the response options for the three types of triptans were still retained in the questionnaire. This decision allowed for the possibility that patients might purchase triptans on their own. Additionally, one neurologist suggested the inclusion of a commonly found medicine in China known as “Toutong Powder”, in which aspirin is the main ingredient.

The relevance, comprehensiveness, and comprehensibility of the all eight items in the questionnaire were agreed upon by the experts, as presented in Table **3**. Each individual item had an I-CVI exceeding the cut-off value of 0.70. The S-CVI_UA_ was 0.75, and the S-CVI_AVE_ was 0.95. While two experts expressed concerns regarding the numerous and unspecific response options related to medicine utilisation in Q1 and Q3, it was ultimately decided to retain all options. This choice was made after thorough deliberation with the aim of capturing a comprehensive picture of medicine utilisation among migraine patients in China.

**Table 3** Content validity of the HARDSHIP healthcare utilisation questionnaire

| **Item No** | **Number in agreement (score ^a^ 3 or 4)** | **I-CVI ^b^** |
| --- | --- | --- |
| 1 | 5 | 0.83 |
| 2 | 6 | 1 |
| 3 | 4 | 0.75 |
| 4 | 6 | 1 |
| 5 | 6 | 1 |
| 6 | 6 | 1 |
| 7 | 6 | 1 |
| 8 | 6 | 1 |
| S-CVI_UA_ ^c^ | | 0.75 |
| S-CVI_AVE_ ^c^ | | 0.95 |
| Note: The I-CVI is calculated as the proportion of experts scoring 3 or 4 among all experts. For the calculation of the S-CVI, there are two common methods, universal agreement (UA) and average (AVE) [13]. S-CVI_UA_ is calculated by dividing the number of items that receive an I-CVI of one by the total number of items in the questionnaire, while S-CVI_AVE_ is the average of the I-CVI for all items in the questionnaire.  ^a^ score 4 = highly relevant and thorough, score 3 = relevant and thorough, score 2 = not so relevant and thorough, score 1 = not relevant and not thorough.  ^b^ I-CVI is calculated as the proportion of experts scoring 3 or 4 among all experts.  ^c^ S-CVI_UA_ is calculated by dividing the number of items that receive an I-CVI of one by the total number of items in the questionnaire, while S-CVI_AVE_ is the average of the I-CVI for all items in the questionnaire. | | |

The pilot test was completed by 38 bank employees who were diagnosed with migraines. During the pilot test, one participant suggested incorporating home-based care for migraines into Q7. Accordingly, this question was modified to “Have you, in the last year, tried any complementary therapies at informal facilities (e.g., home-based or private facilities)?” An additional explanation was added: “In this study, informal facilities refer to healthcare facilities outside of public clinics and hospitals, where most complementary therapies are typically provided.” Concerning the questionnaire format, two participants suggested enlarging the font size of the instructions for filling in the costs in Q6 and Q7, as these instructions could be easily overlooked. Based on feedback from another respondent who was unaware that multiple medicines could be selected in Q1, the instruction regarding multiple choices was clarified and improved for better understanding. Besides these adjustments, all respondents found the instructions and items in the questionnaire clear.

The final version of the HARDSHIP healthcare questionnaire is provided in [Table](#appendixf) **4**. This questionnaire was utilised to investigate healthcare utilisation patterns related to migraines among bank employees in Guizhou province, China.

**Table 4** Final version of the HARDSHIP healthcare utilisation questionnaire

| **No** | **Question** | **Answer** |
| --- | --- | --- |
| 1 | Please look at these lists. Which of these have you used in the last month (no matter prescription or OTC)?  [**Multiple choice**, enter by each medication the number of days on which you used it in the last month.] | □ Nothing at all  **Traditional Chinese patent medicines:**  □ Gastrodia Capsule  □ Zhengtian Pill  □ Lingyangjiao Pill  □ Duliang Soft Capsule  □ Yangxue Qingnao Granule  □ Ershiwuwei Shanhu Wan  □ Tongtian Oral Liquid  □ Tablet of Corydalistuber for Alleviating Pain  □ Seven Leaves Spirit Calmness Tablet  □ Tou Tongning Capsule  **Western medicines:**  □ Toutong Powder  □ Aspirin  □ Acetaminophen (Paracetamol)  □ Ibuprofen  □ Caffeine  □ Paracetamol, aminophenazone, caffeine, and chlorphenamine maleate tablets  □ Weak opioids/opioids  □ Ergot alkaloids  □ Antiemetics  □ Sumatriptan  □ Zolmitriptan  □ Rizatriptan  Number of days ________ |
| 2 | Are there any other medications you have used to treat your headaches in the last month?  [**Multiple choice**] | Name of medications ________  Number of days ________ |

**Table 4** continued

| **No** | **Question** | **Answer** |
| --- | --- | --- |
| 3 | Medications to prevent headaches are usually taken daily. Are you taking any of these now?  [**Multiple choice**] | □Sibelium (Flunarizine)  □ β1-receptor antagonists  □ Antiepileptics  □ Antidepressants  □ Type A botulinum toxin  □ Others ________(name)  ________how long taken |
| 4 | Have you had formal professional advice about your headaches in the last year? Who from, and how many times?  [**Multiple choice**] | □ No one  □ Public clinics  □ Public primary-level hospitals  □ Public secondary-level hospitals  □ Public tertiary-level hospitals  □ Public TCM hospitals  □ Private facilities  Number of times ________ |
| 5 | Because of your headaches, have you had any of these tests in the last year?  [**Multiple choice**] | □ CT scan  □ MRI  □ TCD  □ Electroencephalography  □ Others________(name) |
| 6 | Have you, in the last year, tried any complementary therapies at **public facilities**?  [**Multiple choice**] | □ Acupuncture ________ (expenses)  □ moxibustion ________ (expenses)  □ cupping _____(expenses)  □ Tui Na_____(expenses)  □ Herbology_____(expenses)  □ Others______(name) _____(expenses) |

**Table 4** continued

| **No** | **Question** | **Answer** |
| --- | --- | --- |
| 7 | Have you, in the last year, tried any complementary therapies at **informal facilities** (e.g., home-based or private facilities)?  [**Multiple choice**]  In this study, informal facilities refer to healthcare facilities outside of public clinics and hospitals, where most complementary therapies are typically provided. | □ Acupuncture  □ Herbology  □ Tui Na  □ Cupping  □ Moxibustion  □ Others________(name)  **Expense/year** ________ |
| Abbreviations: OTC, Over-the-counter; TCM, Traditional Chinese Medicine; CT, Computed Tomography; MRI, Magnetic Resonance Imaging; TCD, Transcranial Doppler ultrasonography.  Note: Tui Na refers to Chinese massage therapy. | | |

**References**

1. World Health Organization (2015) People's Republic of China health system review.

2. Liu R, Yu S, He M, Zhao G, Yang X, Qiao X, et al (2013) Health-care utilization for primary headache disorders in China: A population-based door-to-door survey. J Headache Pain 14: 47. doi:10.1186/1129-2377-14-47.

3. Li X, Zhou J, Tan G, Wang Y, Ran L, Chen L (2012) Diagnosis and treatment status of migraine: A clinic-based study in China. J Neurol Sci 315(1): 89-92. doi:10.1016/j.jns.2011.11.021.

4. Yu S, Zhang Y, Yao Y, Cao H (2020) Migraine treatment and healthcare costs: Retrospective analysis of the China Health Insurance Research Association (CHIRA) database. J Headache Pain 21: 53. doi:10.1186/s10194-020-01117-2.

5. Luo N, Qi W, Zhuang C, Di W, Lu Y, Huang Z, et al (2014) A satisfaction survey of current medicines used for migraine therapy in China: Is Chinese patent medicine effective compared with Western medicine for the acute treatment of migraine? Pain Med 15(2): 320. doi:10.1111/pme.12277.

6. Chinese Neurologists Association (2022) 中国偏头痛诊治指南 (2022版). [Guidelines for the diagnosis and treatment of migraine in China (2022 Edition)]. Chinese Journal of Pain Medicine 28(12): 881-898. Retrieved from <https://kns.cnki.net/kcms2/article/abstract?v=6xaVI2TORM3eFtmmRNdT4iXgrnTEdioULPsK7k2Vgq-27rrt1GwrrA3SLnBO1ba-T-mIGxZc0n9rLodAIS_c_fp-yH04ERa9yylhL2zEQ9euT9OR2a93_o0FmIqld6PMpXSr_ViB2odZmveLFN9Bng==&uniplatform=NZKPT&language=CHS>

7. Evers S, Áfra J, Frese A, Goadsby PJ, Linde M, May A, et al (2009) EFNS guideline on the drug treatment of migraine – revised report of an EFNS task force. Eur J Neurol 16(9): 968-981. doi:10.1111/j.1468-1331.2009.02748.x.

8. Ailani J, Burch RC, Robbins MS, the Board of Directors of the American Headache Society (2021) The American Headache Society Consensus Statement: Update on integrating new migraine treatments into clinical practice. Headache 61(7): 1021-1039. doi:10.1111/head.14153.

9. Gao C-Y, Zhang Y-Y, Wu B-S, Liu Y. (2023). 中国偏头痛中西医结合防治指南（2022年）. *Chin J Integr Med, 43*(5), 517-526. Retrieved from <https://kns.cnki.net/kcms2/article/abstract?v=6xaVI2TORM0AVdwiaS35vWQyhvqHdXWBF6_fqYFp0_lINEv-NApXjzxhMBJ-4qljYSN4fhmb4ZKlwu2jRP6yyDkRtKKxRuTdxASrR7eaI8dkdSjD4Mh9NzAMmLfSzAXhJfuoe64uY2xa-jRR-jHRww==&uniplatform=NZKPT&language=CHS>

10. Katsarava Z, Mania M, Lampl C, Herberhold J, Steiner TJ (2018) Poor medical care for people with migraine in Europe - evidence from the Eurolight study. J Headache Pain 19: 10. doi:10.1186/s10194-018-0839-1.

11. McQuade JL, Meng Z, Chen Z, Wei Q, Zhang Y, Bei W, et al (2012) Utilization of and Attitudes towards Traditional Chinese Medicine Therapies in a Chinese Cancer Hospital: A Survey of Patients and Physicians. Evid Based Complement Alternat Med 2012: 504507. doi:10.1155/2012/504507.

12. Sousa VD, Rojjanasrirat W (2011) Translation, adaptation and validation of instruments or scales for use in cross-cultural health care research: a clear and user-friendly guideline. J Eval Clin Pract 17(2): 268-274. doi:10.1111/j.1365-2753.2010.01434.x.

13. Polit DF, Beck CT, Owen SV (2007) Is the CVI an acceptable indicator of content validity? Appraisal and recommendations. Res Nurs Health 30(4): 459-467. doi:10.1002/nur.20199.

14. Beaton DE, Bombardier C, Guillemin F, Ferraz MB (2000) Guidelines for the process of cross-cultural adaptation of self-report measures. Spine (Phila Pa 1976) 25(24): 3186-3191. doi:10.1097/00007632-200012150-00014.

1. With the advancement of TCM, Chinese herbal tonics have evolved into what are known as traditional Chinese patent medicines. These medicines are widely employed in clinical practice in China and are available in various forms like pills, capsules, or syrups. [↑](#footnote-ref-1)
