# Supplementary material for: Healthcare utilisation and economic burden of migraines among bank employees in China: a probabilistic modelling study
Source: J Headache Pain. 2024 Apr 19;25(1):60. doi: 10.1186/s10194-024-01763-w (PMC11027248; doi:10.1186/s10194-024-01763-w)
Supplement: Supplementary file 2 — Additional file 2: Supplementary Material 2. Unit costs of healthcare resources in China (in 2022 USD). [file 10194_2024_1763_MOESM2_ESM.docx]

**Unit costs of healthcare resources in China (in 2022 USD)**

1. **Unit costs of outpatient consultations at public facilities**

The healthcare service system in China operates on a fee-for-service basis, where service charges are applied [1]. The majority of healthcare services are co-paid by basic health insurance schemes and private funding sources [2]. This implies that service charges, regardless of the payer, constitute the primary source of financing for healthcare services in China. In this context, the fee for an outpatient consultation can be considered the unit cost for a Cost-of-Illness (COI) study. Moreover, in China, the tariff for each healthcare service provided at public facilities is officially standardised by each Chinese province, based on the types of public healthcare facilities (clinics, primary-level hospitals, secondary-level hospitals, and tertiary-level hospitals). This is a result of China’s fiscal revenue and expenditure being decentralised, allowing local governments to determine health service fees [2]. In this study, the survey on healthcare utilisation related to migraines was conducted in Guizhou province, China. Accordingly, the 2022 healthcare services tariff of Guizhou province was utilised [3].

In the 2022 healthcare services tariff of Guizhou province, the fee for a consultation at public facilities consisted of four components: (1) Registration Fee, (2) Reservation Fee, (3) Consultation Fee, and (4) Senior Premium. Accordingly, the fee for a consultation = (1) Registration Fee + (2) Reservation Fee + (3) Consultation Fee + (4) Senior Physician Premium.

The first three components, the (1) Registration Fee, (2) Reservation Fee, and (3) Consultation Fee, were directly sourced from the 2022 healthcare services tariff of Guizhou province. The last component, (4) Senior Physician Premium, required special consideration as it is incurred by consultations with senior physicians. In the 2022 healthcare services tariff of Guizhou province, senior physicians include deputy chief physicians, chief physicians, and specialists. In China, physicians progress through seniority levels, starting from resident physicians and progressing to attending physicians, deputy chief physicians, and chief physicians [4]. Some chief physicians are recognised as specialists in the specific medical fields in which they practice.

In general, clinics and primary-level hospitals have only attending physicians providing primary care. At these facilities, patients are not charged for Senior Doctor Premium. Therefore, the unit cost of an outpatient consultation at public clinics or primary-level hospitals was calculated as the sum of the (1) Registration Fee, (2) Reservation Fee, and (3) Consultation Fee.

Secondary- and tertiary-level hospitals employ deputy chief physicians and chief physicians (including specialists) in addition to attending physicians. Consultations with these senior physicians incur an additional fee known as the Senior Physician Premium: in Chinese yuan (CNY), ¥5.0 for deputy chief physicians, ¥6.0 for chief physicians, and ¥10.0 for specialists, as sourced from the 2022 healthcare services tariff of Guizhou province. In this study, the number of outpatient consultations with these senior physicians was assumed to be proportional to their availability. Therefore, at secondary- and tertiary-level hospitals, the Senior Physician Premium was determined as a weighted average Senior Physician Premium considering the proportions of deputy chief physicians, chief physicians, and specialists among all physicians. According to the National Health Commission of the People’s Republic of China [5], deputy chief physicians and chief physicians (including specialists) constituted 18.4% and 8.1% of all physicians, respectively. Based on the above information, the weighted average Senior Physician Premium at secondary- and tertiary-level hospitals was calculated as 18.4% × ¥5.0 + 8.1% × [(¥6.0 + ¥10.0) ÷ 2] = ¥1.6. Combining the Registration Fee, Reservation Fee, and Consultation Fee, the unit cost of an outpatient consultation at public clinics, primary-level hospitals, secondary-level hospitals, and tertiary-level hospitals was calculated as ¥2.5, ¥4.5, ¥8.1, and ¥8.1, respectively, as detailed in Table **1**.

Another type of public hospitals in China is the Traditional Chinese Medicine (TCM) hospitals. Public TCM hospitals can operate at primary, secondary, or tertiary levels; therefore, the fee for an outpatient consultation at public TCM hospitals could be estimated as a weighted average fee. In this work, the distribution of public TCM hospitals was assumed to be proportional to the number of public primary-level (46.6%), secondary-level (40.6%) and tertiary-level (12.8%) hospitals. The proportional data were obtained from the National Health Commission of the People’s Republic of China [6]. Accordingly, the unit cost of an outpatient consultation at public TCM hospitals was estimated as a weighted average cost, calculated as 46.6% × ¥4.5 + 40.6% × ¥8.1 + 12.8% × ¥8.1 = ¥6.4 (Table **1**).

Table **1** provides a breakdown of the estimated unit costs by public healthcare facility type. When converted to United States dollars (USD), the unit cost of an outpatient consultation at public clinics, public primary-level hospitals, public secondary-level hospitals, public tertiary-level hospitals, or public TCM hospitals was estimated to be $0.7, $1.3, $2.3, $2.3, and $1.8, respectively.

**Table 1** Estimation of the unit costs of outpatient consultations at public facilities in Guizhou province, China (in 2022 USD)

| **Cost item** | **Proportion of physicians** ^a^ | **Clinics** | **Primary-Level**  **hospitals** | **Secondary-Level**  **hospitals** | **Tertiary-Level**  **hospitals** | **TCM**  **hospitals** |
| --- | --- | --- | --- | --- | --- | --- |
| **Distribution of hospitals ^b^** | | N/A | 46.6% | 40.6% | 12.8% | N/A |
| (1) Registration Fee (¥) **^c^** | | 0.5 | 0.5 | 0.5 | 0.5 | N/A |
| (2) Reservation Fee (¥) **^c^** | | 0 | 2.0 | 3.0 | 3.0 | N/A |
| (3) Consultation Fee **^c^** | | 2.0 | 2.0 | 3.0 | 3.0 |  |
| (4) Senior Physician Premium **^c^** | | | | | | |
| Deputy chief physician visit (¥) | 18.4% ^a^ | N/A ^e^ | N/A ^e^ | 5.0 **^c^** | 5.0 **^c^** | N/A |
| Chief physician visit (¥) | 8.1% for the combined percentage of chief physicians and specialists ^a^ | N/A ^e^ | N/A ^e^ | 6.0 **^c^** | 6.0 **^c^** | N/A |
| Specialist visit (¥) |  | N/A ^e^ | N/A ^e^ | 10.0 **^c^** | 10.0 **^c^** | N/A |
| **Weighted average consultation fee (¥)** | | **N/A** ^e^ | **N/A** ^e^ | **1.6 ^f^** | **1.6 ^f^** | **N/A** |
| **Cost of an outpatient consultation (¥) ^d^** | | **2.5 ^e^** | **4.5 ^e^** | **8.1 ^g^** | **8.1 ^g^** | **6.4 ^h^** |
| **Cost of an outpatient consultation (converted to USD ^i^)** | | **0.7** | **1.3** | **2.3** | **2.3** | **1.8** |
| Abbreviations: TCM, Traditional Chinese Medicine; USD, United States dollars; CNY, Chinese yuan; N/A, Not Applicable.  ^a^ According to the National Health Commission of the People’s Republic of China (2022), deputy chief physicians and chief physicians (including specialists) constituted 18.4% and 8.1% of all physicians, respectively.  ^b^ According to the National Health Commission of the People’s Republic of China (2023), public primary-level hospitals represented 46.6% of the total public hospitals, public secondary-level hospitals represented 40.6%, and public tertiary-level hospitals represented 12.8%.  ^c^ The (1) Registration Fee, (2) Reservation Fee, and (3) Consultation Fee were directly sourced from the 2022 healthcare services tariff of Guizhou province. The (4) Senior Physician Premium required special consideration as it is incurred by consultations with senior physicians. In the 2022 healthcare services tariff of Guizhou province, senior physicians include deputy chief physicians, chief physicians, and specialists. Consultations with these senior physicians incur an additional fee known as the Senior Physician Premium: in CNY, ¥5.0 for deputy chief physicians, ¥6.0 for chief physicians, and ¥10.0 for specialists, as sourced from the 2022 healthcare services tariff of Guizhou province.  ^d^ Fee for an outpatient consultation = (1) Registration Fee + (2) Reservation Fee + (3) Consultation Fee + (4) Senior Physician Premium  ^e^ In general, clinics and primary-level hospitals have only attending physicians providing primary care. At these facilities, patients are not charged for Senior Doctor Premium. Therefore, the unit cost of an outpatient consultation at public clinics or primary-level hospitals was calculated as the sum of the (1) Registration Fee, (2) Reservation Fee, and (3) Consultation Fee.  Unit cost of an outpatient consultation at public clinics = ¥0.5 + ¥0 + ¥2.0 = ¥2.5  Unit cost of an outpatient consultation at public primary-level hospitals = ¥0.5 + ¥2.0 + ¥2.0 = ¥4.5  ^f^ In this study, the number of outpatient consultations with senior physicians was assumed to be proportional to their availability. Therefore, at secondary- and tertiary-level hospitals, the Senior Physician Premium was determined as a weighted average Senior Physician Premium considering the proportions of these senior physicians among all physicians. According to the National Health Commission of the People’s Republic of China (2022), deputy chief physicians and chief physicians (including specialists) constituted 18.4% and 8.1% of all physicians, respectively. Based on the above information, the weighted average Senior Physician Premium at secondary- or tertiary-level hospitals was calculated as 18.4% × ¥5.0 + 8.1% × [(¥6.0 + ¥10.0) ÷ 2] = ¥1.6.  ^g^ Unit cost of an outpatient consultation at public secondary- or tertiary-level hospitals = ¥0.5 + ¥3.0 + ¥3.0 +¥1.6 = ¥8.1  ^h^ In this work, the distribution of public TCM hospitals was assumed to be proportional to the number of public primary-level (46.6%), secondary-level (40.6%) and tertiary-level (12.8%) hospitals. As a result, the unit cost of an outpatient consultation at public TCM hospitals was estimated as a weighted average cost = 46.6% × ¥4.5 + 40.6% × ¥8.1 + 12.8% × ¥8.1 = ¥6.4  ^i^ All CNY costs were adjusted to 2022 USD using Gross Domestic Product (GDP) deflator indexes and purchasing power parity values. | | | | | | |

1. **Unit costs of diagnostic tests at public facilities**

The healthcare service system in China operates on a fee-for-service basis, where service charges are applied [1]. The majority of healthcare services are co-paid by basic health insurance schemes and private funding sources [2]. This implies that service charges, regardless of the payer, constitute the primary source of financing for healthcare services in China. In this context, the fee for a diagnostic test can be considered the unit cost for a COI study. Moreover, in China, the tariff for each healthcare service provided at public facilities is officially standardised by each Chinese province, based on the types of public healthcare facilities (clinics, primary-level hospitals, secondary-level hospitals, and tertiary-level hospitals). This is a result of China’s fiscal revenue and expenditure being decentralised, allowing local governments to determine health service fees [2]. In this study, the survey on healthcare utilisation related to migraines was conducted in Guizhou province, China. Accordingly, the 2022 healthcare services tariff of Guizhou province was utilised [3].

Previous studies reported that computed tomography (CT) scan, magnetic resonance imaging (MRI), transcranial Doppler ultrasonography (TCD), and electroencephalography (EEG) tests are utilised by migraine patients in China [7-9]. However, these diagnostic tests are not available in clinics due to the lack of required medical equipment. Instead, they are primarily conducted at hospitals, including primary-, secondary-, tertiary-level, and TCM hospitals. The fee data for CT scan, MRI, TCD, and electroencephalography tests at public primary-level hospitals, public secondary-level hospitals, and public tertiary-level hospitals were directly sourced from the 2022 healthcare services tariff of Guizhou province, shown in Table **2**.

Another type of public hospitals in China is the TCM hospitals. Public TCM hospitals can operate at primary, secondary, or tertiary levels; therefore, the fee for a diagnostic test at public TCM hospitals could be estimated as a weighted average fee. In this work, the distribution of public TCM hospitals was assumed to be proportional to the number of public primary-level (46.6%), secondary-level (40.6%) and tertiary-level (12.8%) hospitals. The proportional data were obtained from the National Health Commission of the People’s Republic of China [6]. Accordingly, the unit cost of a diagnostic test at public TCM hospitals was estimated as a weighted average cost (see Table **2**).

**Table** **2** Estimation of the unit costs of diagnostic tests at public facilities in Guizhou province, China (in 2022 USD)

| **Test** | **Primary-Level hospitals (USD** ^a^**)** | **Secondary-Level hospitals (USD** ^a^**)** | **Tertiary-Level hospitals (USD** ^a^**)** | **TCM hospitals**  **(USD** ^a^**)** |
| --- | --- | --- | --- | --- |
| Distribution of the three types of hospitals ^b^ | 46.6% | 40.6% | 12.8% | Proportional to the number of the three types of hospitals ^d^ |
| CT scan ^c^ | 31.3 | 36.9 | 42.6 | 35.0 ^d^ |
| MRI ^c^ | 170.5 | 204.5 | 227.3 | 191.6 ^d^ |
| TCD ^c^ | 22.7 | 28.4 | 31.3 | 26.1 ^d^ |
| Electroencephalography ^c^ | 7.1 | 8.5 | 9.9 | 8.0 ^d^ |

Abbreviations: TCM, Traditional Chinese Medicine; CT, Computed Tomography; MRI, Magnetic Resonance Imaging; TCD, Transcranial Doppler ultrasonography; USD, United States dollars.

^a^ All CNY costs were adjusted to 2022 USD using GDP deflator indexes and purchasing power parity values.

^b^ According to the National Health Commission of the People’s Republic of China (2023), public primary-level hospitals represented 46.6% of the total public hospitals, public secondary-level hospitals represented 40.6%, and public tertiary-level hospitals represented 12.8%.

^c^ Previous studies reported that CT scan, MRI, TCD, and electroencephalography tests are utilised by migraine patients in China [7-9]. However, these diagnostic tests are not available in clinics due to the lack of required medical equipment. Instead, they are primarily conducted at hospitals, including primary-, secondary-, tertiary-level, and TCM hospitals. The fee data for CT scan, MRI, TCD, and electroencephalography tests at public primary-level hospitals, public secondary-level hospitals, and public tertiary-level hospitals were directly sourced from the 2022 healthcare services tariff of Guizhou province.

^d^ In this work, the distribution of public TCM hospitals was assumed to be proportional to the number of public primary-level (46.6%), secondary-level (40.6%), and tertiary-level (12.8%) hospitals. Accordingly, the unit cost of a diagnostic test at public TCM hospitals was estimated as a weighted average cost:

Unit cost of a CT scan at TCM hospitals: 46.6% × $31.3 + 40.6% × $36.9 + 12.8% × $42.6 = $35.0

Unit cost of an MRI at TCM hospitals: 46.6% × $170.5 + 40.6% × $204.5 + 12.8% × $227.3 = $191.6

Unit cost of a TCD at TCM hospitals: 46.6% × $22.7 + 40.6% × $28.4 + 12.8% × $31.3 = $26.1

Unit cost of an electroencephalography at TCM hospitals: 46.6%% ×$7.1 + 40.6% × $8.5 + 12.8% × $9.9 = $8.0

1. **Unit costs of outpatient visits at private facilities**

In China, healthcare charges at private facilities in China lack an official standard. Nevertheless, the National Health Commission of the People’s Republic of China compiles an all-cause average fee per outpatient visit, encompassing outpatient consultations and diagnostic tests, across all providers annually. This commission is responsible for formulating and implementing health policies in China [10]. For this study, the unit cost of an outpatient visit at private facilities was estimated based on the reported average fee of $97.4 in 2022 USD [6].

1. **Daily costs of medicines**

The HARDSHIP healthcare utilisation questionnaire simplifies questions for respondents by focusing on the number of medication days rather than detailed dosage specifics. To estimate the costs of each medicine, the reported annual number of medication days for each medicine can be multiplied by the corresponding daily cost.

While it is acknowledged that the daily cost of a medicine varies among patients with different conditions due to differing daily doses, an average daily cost can still be calculated based on the recommended daily dosage indicated on the medicine label. The daily cost of a medicine was calculated by multiplying the recommended daily dosage by medicine type with the wholesale price per medicine unit.

The daily dosage recommendations for migraines, as indicated on the label of each medicine, were strictly followed. The daily costs of medicines in this study were estimated based on wholesale prices instead of retail prices. This choice was made because wholesale prices reflect the opportunity cost of a resource, representing the value forgone if this resource is not optimally allocated [11].

For over-the-counter (OTC) medicines, both brand-name and generic products are available. The study opted for the lowest wholesale prices, as evidence indicates that consumers generally have confidence in the safety and effectiveness of both brand-name and generic products and tend to prefer the most economical options [12,13]. Similarly, the lowest wholesale prices for prescription medicines were adopted. This decision aligns with China’s National Centralised Drug Procurement Policy, which determines the selling prices of medicines in healthcare facilities based on the lowest bid or negotiated costs, without including a profit margin [14,15].

The wholesale prices per medicine unit were sourced from a publicly available source “Medicine Price Checker” (<http://www.china-yao.com/>) in 2022. The daily costs of medicines presented in Table **3** are kept in CNY because these costs are too small. The total costs for medicines would be later converted into USD.

**Table 3** Estimation of the daily costs of migraine medicines in China (in 2022 CNY)

| **Medicine** | | **Company** | **Daily cost ^b^ (CNY)** |
| --- | --- | --- | --- |
| **Acute medicines** | | | |
|  | **Traditional Chinese patent medicines ^a^** | | |
|  | Gastrodia Capsule | 通化金马药业集团股份有限公司Tonghua Golden-Horse | 0.5 ^b^ |
|  | Zhengtian Pill | 三九医药股份有限公司China Resources Sanjiu | 3.9 |
|  | Tou tong-ning Capsule | 陕西步长制药有限公司Shaanxi Buchang | 6.3 |
|  | Duliang soft Capsule | 重庆华森制药有限公司Chongqing Pharscin | 8.1 |
|  | Yangxue Qingnao Granule | 东莞万成制药有限公司Dongguan Wancheng | 5.1 |
|  | Lingyangjiao Pills | 东北虎药业股份有限公司Northeast Tiger | 7.6 |
|  | Ershiwuwei Shanhu Wan | 西藏金珠雅砻藏药有限责任公司Along Tibet | 6.0 |
|  | Tongtian Oral Liquid | 重庆赛诺生物药业有限公司Chongqing Sino | 6.8 |
|  | Tablet of Corydalistuber for Alleviating Pain | 重庆东方药业股份有限公司Chongqing Dongfang | 0.7 |
|  | Seven Leaves Spirit Calmness Tablet | 昆明大观制药厂Kunming Daguan | 0.3 |
|  | 999 Ganmao Ling Keli | 药都制药集团股份有限公司Yaodu | 1.1 |
|  | **Western medicines** | | |
|  | Toutong Powder | 重庆科瑞制药有限责任公司Chongqing Kerui | 0.1 |
|  | Aspirin | 青岛黄海制药有限责任公司Qingdao Huanghai | 0.0 ^d^ |
|  | Ibuprofen | 山东方明药业集团股份有限公司Shandong Fangming | 0.1 |
|  | Naproxen | 成都通德药业有限公司Chengdu Tongde | 0.8 |
|  | Paracetamol, aminophenazone, caffeine, and chlorphenamine maleate tablets | 神威药业有限公司Shenwei | 0.9 |
|  | Diclofenac Sodium Sustained Release Capsule | 深圳致君制药有限公司Zhijun | 1.0 |
|  | Acetaminophen (Paracetamol) | 汕头金石制药有限公司Shantou Jinshi | 0.4 |
|  | Ibuprofen and codeine | 中国医药集团总公司Sinopharm | 5.7 |
|  | Tramadol | 黑龙江龙桂制药有限公司Heilongjiang Longgui | 2.6 |
|  | Nicergoline | 辉瑞制药有限公司Pfizer | 3.3 |
|  | Ergotamine Tartrate/caffeine | 哈尔滨泰华药业股份有限公司Harbin Taihua | 11.6 |
|  | Barbiturates | 上海信谊药厂有限公司Shanghai Sine | 0.1 |

**Table 3** continued

| **Medicine** | | **Company** | **Daily cost ^b^ (CNY)** |
| --- | --- | --- | --- |
|  | Metoclopramide | 山西云鹏药业有限公司Yunpeng | 0.1 |
|  | Domperidone | 江西捷众生物化学有限公司Jiangxi Jiezhong | 0.7 |
|  | Glucocorticoids | 浙江仙琚制药股份有限公司Zhejiang Xianju | 0.1 |
|  | Mannitol injection | 山东齐都药业有限公司Shandong Qidu | 1.4 |
|  | Sumatriptan ^c^ | 天津华津制药厂Tianjin Huajin | 21.0 |
|  | Zolmitriptan ^c^ | 四川省旭晖制药有限公司Sichuan Xuhui | 16.0 |
|  | Rizatriptan ^c^ | 四川梓橦宫药业股份有限公司Sichuan Zitonggong | 42.5 |
|  | Japan EVE QUICK Painkiller ^c^ | SSP | 6.4 |
| **Preventive medicines** | | | |
|  | Sibelium (Flunarizine) | 山西津华晖星制药有限公司Shanxi Jinhuahuixing | 0.1 |
|  | Lomefloxacin | 南京长澳制药有限公司Nanjing Chang'ao | 2.7 |
|  | β1-receptor antagonists | 天津市中央药业有限公司THE Central | 0.1 |
|  | Magnesium valproate | 湖南省湘中制药有限公司Hunan Xiangzhong | 1.0 |
|  | Sodium valproate | 湖南省湘中制药有限公司Hunan Xiangzhong | 0.4 |
|  | Topiramate | 西安杨森制药有限公司Xian Janssen | 1.3 |
|  | Gabapentin | 江苏恩华药业股份有限公司Jiangsu Nhwa | 1.2 |
|  | Vitamin B_2_ | 湖北广济药业股份有限公司Hubei Guangji | 0.0 ^d^ |
|  | Coenzyme Q10 | 上海普康药业有限公司Shanghai Pukang | 0.6 |
|  | Candesartan Cilexetil | 广州白云山天心制药股份有限公司Guangzhou Baiyunshan Tianxin | 1.7 |
|  | Prednisone | 浙江仙琚制药股份有限公司Zhejiang Xianju | 0.1 |
|  | Duliang Soft Capsule | 重庆华森制药有限公司Chongqing Pharscin | 8.1 |
|  | Yangxue Qingnao Granule | 东莞万成制药有限公司Dongguan Wancheng | 5.1 |

Abbreviations: CNY, Chinese yuan; SSP, SSP Co., Ltd; TCM, Traditional Chinese Medicine.

^a^ With the advancement of TCM, Chinese herbal tonics have evolved into what are known as traditional Chinese patent medicines. These medicines are widely employed in clinical practice in China and are available in various forms like pills, capsules, or syrups.

^b^ Daily cost by medicine type = Recommended daily dosage indicated on medicine label × Wholesale price per medicine unit

The daily dosage recommendations for migraines, as indicated on the label of each medicine, were strictly followed. The wholesale prices per medicine unit were sourced from a publicly available source “Medicine Price Checker” (<http://www.china-yao.com/>) in 2022.

Taking Gastrodia Capsule as an example, the daily dosage recommended on the label is 18 grams, and the cheapest wholesale price per gram is ¥0.03, sourced from the “Medicine Price Checker” (http://www.china-yao.com/). Consequently, the daily cost of Gastrodia Capsule was calculated as follows: 18 grams × ¥0.03 per gram = ¥0.54 (rounded to ¥0.5 when expressed in one decimal place).

^c^ According to expert interviews conducted during the content validation process for the HARDSHIP healthcare utilisation questionnaire (as detailed in [Supplementary](#s5422b) Material [**1**](Supplementary%20Material%201.docx)), the accessibility of these medication is limited in most hospitals and pharmacies in China. This study used the prices from the leading online retailers for these medicines.

^d^ The daily costs of Aspirin and Vitamin B2, although rounded to ¥0.0 in this table, were actually both ¥0.024.

1. **Per-Patient costs for complementary therapies**

Given the unstandardised nature of complementary therapies and the personalised treatment regimens administered to each patient, determining the precise costs of a single session of these therapies poses a challenge. To overcome this, this study used data obtained from our survey. Specifically, the respondents were queried about the expenses they paid for each type of migraine-related complementary therapy at either public or informal facilities in 2022. In this study, informal facilities refer to healthcare facilities outside of public clinics and hospitals, where the majority of complementary therapies are provided. Given the skewed distributions of the cost data provided by the respondents, this analysis opted to present median values, as shown in Table **4**.

**Table 4** Estimation of the per-patient costs for complementary therapies in Guizhou province, China (in 2022 USD)

| **Complementary therapy** | **Median cost ^a^ (USD ^b^)** |
| --- | --- |
| **At public facilities** | |
| Acupuncture | 15.4 |
| Moxibustion | 39.6 |
| Cupping | 14.0 |
| Tui Na (Chinese massage therapy) | 82.7 |
| Chinese herbal medicine | 182.8 |
| **At informal facilities** | |
| Acupuncture | 85.0 |
| Moxibustion | 24.8 |
| Cupping | 3.4 |
| Tui Na (Chinese massage therapy) | 142.0 |
| Chinese herbal medicine | 273.8 |
| Others | 28.4 |

Abbreviation: USD, United States dollars; CNY, Chinese yuan.

^a^ Given the skewed distributions of the cost data provided by the respondents, this analysis opted to present median values.

^b^ All CNY costs were adjusted to 2022 USD using GDP deflator indexes and purchasing power parity values.

**References**

1. Poulos C, Riewpaiboon A, Stewart JF, Clemens J, Guh S, Agtini M, et al (2011) Cost of illness due to typhoid fever in five Asian countries. Trop Med Int Health 16(3): 314-323. doi:10.1111/j.1365-3156.2010.02711.x.

2. World Health Organization (2015) People's Republic of China health system review.

3. Guizhou Provincial People's Government (2022) Unit costs for healthcare services in Guizhou province. <https://ylbzj.guizhou.gov.cn/zfxxgk/fdzdgknr/zcfg_5623824/202107/t20210723_69216574.html>. Accessed

4. Zhang L (2014) Medical hierarchy in professional ranking and its implications in China. China Health Review 5(2): 16-17. Retrieved from <https://review.chpams.org/article/view/15196>

5. National Health Commission of the People’s Republic of China (2022) 2022 Chinese Health Statistics Yearbook. Peking Union Medical College Press.

6. National Health Commission of the People’s Republic of China (2023) Statistical Communiqué of the People's Republic of China on the 2022 National Healthcare Development. <http://www.nhc.gov.cn/guihuaxxs/s3585u/202309/6707c48f2a2b420fbfb739c393fcca92/files/9b3fddc4703d4c9d9ad399bcca089f03.pdf>. Accessed

7. Li X, Zhou J, Tan G, Wang Y, Ran L, Chen L (2012) Diagnosis and treatment status of migraine: A clinic-based study in China. J Neurol Sci 315(1): 89-92. doi:10.1016/j.jns.2011.11.021.

8. Liu R, Yu S, He M, Zhao G, Yang X, Qiao X, et al (2013) Health-care utilization for primary headache disorders in China: A population-based door-to-door survey. J Headache Pain 14: 47. doi:10.1186/1129-2377-14-47.

9. Yu S, Zhang Y, Yao Y, Cao H (2020) Migraine treatment and healthcare costs: Retrospective analysis of the China Health Insurance Research Association (CHIRA) database. J Headache Pain 21: 53. doi:10.1186/s10194-020-01117-2.

10. National Health Commission of the People’s Republic of China (2022) What we do. <http://en.nhc.gov.cn/2018-09/22/c_74499.htm>. Accessed

11. Drummond MF, Sculpher MJ, Claxton K, Stoddart GL, Torrance GW (2015) Methods for the economic evaluation of health care programmes (4th ed.). Oxford University Press, New York, United States.

12. Håkonsen H, Wängberg M, Alani D, Hedenrud T (2020) Generic versus brand-name over-the-counter analgesics: knowledge and attitudes among Swedish pharmacy customers. Journal of Pharmaceutical Policy and Practice 13: 60. doi:10.1186/s40545-020-00269-5.

13. Kohli E, Buller A (2013) Factors influencing consumer purchasing patterns of generic versus brand name over-the-counter drugs. South Med J 106(2): 155-160. doi:10.1097/SMJ.0b013e3182804c58.

14. Hu J, Mossialos E (2016) Pharmaceutical pricing and reimbursement in China: When the whole is less than the sum of its parts. Health Policy 120(5): 519-534. doi:10.1016/j.healthpol.2016.03.014.

15. Peng Z, Zhan C, Ma X, Yao H, Chen X, Sha X, et al (2021) Did the universal zero-markup drug policy lower healthcare expenditures? Evidence from Changde, China. BMC Health Serv Res 21: 1-11. doi:10.1186/s12913-021-07211-8.
