# Supplementary material for: Healthcare utilisation and economic burden of migraines among bank employees in China: a probabilistic modelling study
Source: J Headache Pain. 2024 Apr 19;25(1):60. doi: 10.1186/s10194-024-01763-w (PMC11027248; doi:10.1186/s10194-024-01763-w)
Supplement: Supplementary file 3 — Additional file 3: Supplementary Material 3. Model inputs and data sources for estimating the economic burden of migraines among bank employees. [file 10194_2024_1763_MOESM3_ESM.docx]

**Model inputs and data sources for estimating the economic burden of migraines among bank employees**

Table **1** offers a list of model inputs and data sources for the point estimation. In cases where input parameters exhibited skewed distributions, median values were employed in this analysis.

The joint uncertainty across all input parameters was evaluated by probabilistic sensitivity analyses. Input parameters were incorporated into the probabilistic decision-analytic model using a combination of point estimates and probability distributions. According to the results of the one-way sensitivity analysis (refer to Fig. [4](main%20manuscript.docx) in the main manuscript), parameters including the unit costs of healthcare resources, the annual number of outpatient consultations or diagnostic tests for a patient, proportions of diagnostic tests, the annual days a patient was on medication, and the proportions of medicine use exhibited minimal impact on the total costs. Accordingly, these parameters were deemed less important and were assigned a point-estimate value rather than a distribution.

Other input parameters were incorporated as a probability distribution. This study followed the guidelines provided by Drummond et al. (2015) in selecting assumed distributions. Specifically, beta distributions were used for proportions, while log-normal distributions were used for days and costs [1]. The incorporation of all input parameters for the probabilistic sensitivity analyses is also detailed in Table **1**.

**Table 1** Data sources for the model inputs and their incorporation for probabilistic sensitivity analysis

| **Input parameter** | **Data source** | **Value/Median** | **Data incorporation for** **probabilistic sensitivity analyses ^a^** |
| --- | --- | --- | --- |
| **General parameter** |  |  |  |
| Population | Guizhou Provincial Bureau of Statistics [2] | 130.4 thousand | Point estimate |
| Male-to-female ratio | Guizhou Provincial Bureau of Statistics [2] | 50.4% to 49.6% | Point estimate |
| Male prevalence (%) | Wei et al. [3] | 25.0% | Beta  (α = 220; β = 658) |
| Female prevalence (%) | Wei et al. [3] | 29.2% | Beta  (α = 308; β = 747) |
| **Proportion of outpatient consultations (%)** | | | |
| Public clinics ^b^ | Table [1](main%20manuscript.docx) in the main manuscript | 8.7% | Beta (α = 47; β = 480) |
| Public primary-level hospitals ^b^ | Table [1](main%20manuscript.docx) in the main manuscript | 4.7% | Beta (α = 26; β = 502) |
| Public secondary-level hospitals ^b^ | Table [1](main%20manuscript.docx) in the main manuscript | 6.6% | Beta (α = 36; β = 491) |
| Public tertiary-level hospitals ^b^ | Table [1](main%20manuscript.docx) in the main manuscript | 4.7% | Beta (α = 26; β = 502) |
| Public TCM hospitals ^b^ | Table [1](main%20manuscript.docx) in the main manuscript | 3.6% | Beta (α = 20; β = 507) |
| Private facilities ^b^ | Table [1](main%20manuscript.docx) in the main manuscript | 2.1% | Beta (α = 12; β = 515) |
| **Annual no. of outpatient consultations for a patient** | | | |
| Public clinics | Our survey | 2 | Point estimate |
| Public primary-level hospitals | Our survey | 2.1 | Point estimate |
| Public secondary-level hospitals | Our survey | 2 | Point estimate |
| Public tertiary-level hospitals | Our survey | 2 | Point estimate |
| Public TCM hospitals | Our survey | 3 | Point estimate |
| Private facilities | Our survey | 5 | Point estimate |

**Table 1** continued

| **Input parameter** | **Data source** | **Value/Median** | **Data incorporation for** **probabilistic sensitivity analyses ^a^** |
| --- | --- | --- | --- |
| **Proportion of diagnostic tests (%)** | | | |
| CT scan ^c^ | Our survey | 69.6%, 43.9%, 54.1%, and 37.5% at each public hospital ^d^ | Point estimate |
| MRI ^c^ | Our survey | 21.7%, 26.6%, 32.0%, and 29.4% at each public hospital ^d^ | Point estimate |
| TCD ^c^ | Our survey | 14.4%, 16.4%, 32.0%, and 18.4% at each public hospital ^d^ | Point estimate |
| Electroencephalography ^c^ | Our survey | 18.4%, 39.0%, 48.9%, and 18.1% at each public hospital ^d^ | Point estimate |
| **Annual no. of outpatient consultations for a patient** | Assumption ^e^ | 1 | Point estimate |
| **Proportion of medicine use** | Table [2](main%20manuscript.docx) in the main manuscript | By medicine type ^f^ | Point estimate |
| **Annual days a patient was on medication** | Our survey | By medicine type ^f^ | Point estimate |
| **Proportion of complementary therapy use at public facilities (%)** | | | |
| Acupuncture ^g^ | Our survey | 7.0% | Beta (α = 38; β = 488) |
| Moxibustion ^g^ | Our survey | 5.1% | Beta (α = 28; β = 499) |
| Cupping ^g^ | Our survey | 3.8% | Beta (α = 21; β = 506) |
| Tui Na ^g^ | Our survey | 3.9% | Beta (α = 22; β = 504) |
| Chinese herbal medicine ^g^ | Our survey | 4.1% | Beta (α = 22; β = 504) |
| **Proportion of complementary therapy use at informal facilities (%)** | | | |
| Acupuncture ^g^ | Our survey | 7.5% | Beta (α = 40; β = 487) |
| Moxibustion ^g^ | Our survey | 5.9% | Beta (α = 32; β = 495) |
| Cupping ^g^ | Our survey | 3.9% | Beta (α = 22; β = 506) |
| Tui Na ^g^ | Our survey | 8.7% | Beta (α = 47; β = 480) |
| Chinese herbal medicine ^g^ | Our survey | 3.9% | Beta (α = 22; β = 506) |
| Others ^g^ | Our survey | 1.9% | Beta (α = 11; β = 516) |

**Table 1** continued

| **Input parameter** | **Data source** | **Value/Median** | **Data incorporation for** **probabilistic sensitivity analyses ^a^** |
| --- | --- | --- | --- |
| **Unit costs of outpatient consultations, diagnostic tests, daily costs of medicines** | Supplementary Material [2](Supplementary%20Material%202.docx) | By healthcare resource type | Point estimate |
| **Per-patient costs for complementary therapies at public facilities (in 2022 USD)** | | | |
| Acupuncture | Supplementary Material [2](Supplementary%20Material%202.docx) | 15.4 | Log-normal (meanlog = 2.66; sdlog = 2.67) |
| Moxibustion | Supplementary Material [2](Supplementary%20Material%202.docx) | 39.6 | Log-normal (meanlog = 2.49; sdlog = 2.82) |
| Cupping | Supplementary Material [2](Supplementary%20Material%202.docx) | 14.0 | Log-normal (meanlog = 1.75; sdlog = 2.46) |
| Tui Na | Supplementary Material [2](Supplementary%20Material%202.docx) | 82.7 | Log-normal (meanlog = 2.99; sdlog = 2.85) |
| Chinese herbal medicine | Supplementary Material [2](Supplementary%20Material%202.docx) | 182.8 | Log-normal (meanlog = 4.47; sdlog = 2.89) |
| **Per-patient costs for complementary therapies at informal facilities (in 2022 USD)** | | | |
| Acupuncture | Supplementary Material [2](Supplementary%20Material%202.docx) | 85.0 | Log-normal (meanlog = 3.16; sdlog = 3.06) |
| Moxibustion | Supplementary Material [2](Supplementary%20Material%202.docx) | 24.8 | Log-normal (meanlog = 1.97; sdlog = 2.58) |
| Cupping | Supplementary Material [2](Supplementary%20Material%202.docx) | 3.4 | Log-normal (meanlog = 1.82; sdlog = 2.72) |
| Tui Na | Supplementary Material [2](Supplementary%20Material%202.docx) | 142.0 | Log-normal (meanlog = 3.69; sdlog = 2.82) |
| Chinese herbal medicine | Supplementary Material [2](Supplementary%20Material%202.docx) | 273.8 | Log-normal (meanlog = 4.39; sdlog = 2.57) |
| Others | Supplementary Material [2](Supplementary%20Material%202.docx) | 28.4 | Log-normal (meanlog = 2.13; sdlog = 2.44) |

**Table 1** continued

| **Input parameter** | **Data source** | **Value/Median** | **Data incorporation for** **probabilistic sensitivity analyses ^a^** |
| --- | --- | --- | --- |
| **Number of lost workdays over a three-month period for a patient due to migraine** | | | |
| Males | Our survey | 6 | Log-normal (meanlog = 1.99; sdlog = 1.04) |
| Females | Our survey | 2 | Log-normal (meanlog = 1.65; sdlog = 1.02) |
| **Daily wage (in 2022 USD)** | [Main manuscript](main%20manuscript.docx)**,** sourced from our survey | 83.9 | Point estimate |

Abbreviations: Beta, Beta distribution; Log-normal, Log-normal distribution; TCM, Traditional Chinese Medicine; CT, Computed Tomography; MRI, Magnetic Resonance Imaging, TCD, Transcranial Doppler ultrasonography; no., Number.

Note: Tui Na refers to Chinese massage therapy.

^a^ The joint uncertainty across all parameters was evaluated by incorporating them into the decision-analytic model using a combination of point estimates and probability distributions.

^b^ According to Supplementary Material [1](Supplementary%20Material%201.docx), China’s healthcare service delivery system includes both public and private healthcare facilities [4], allowing patients the flexibility to seek healthcare services from various levels of care [5]. China’s public healthcare system comprises clinics, primary-level hospitals, secondary-level hospitals, tertiary-level hospitals and TCM hospitals.

^c^ According to Supplementary Material [1](Supplementary%20Material%201.docx), CT scan, MRI, TCD, and electroencephalography are commonly used among Chinese migraine patients [5-7], as identified through our review.

^d^ Since the unit costs of health services in China are available from an official tariff exclusively for public facilities, the study focused on detailing the outpatient visit pathway within these public healthcare settings (refer to the [main manuscript](main%20manuscript.docx)**).** Moreover, CT scan, MRI, TCD, and electroencephalography tests are not available in clinics due to the lack of required medical equipment. Instead, they are primarily conducted at hospitals, including primary-, secondary-, tertiary-level, and TCM hospitals. This reflects common treatment practices where, if patients do not receive a definitive diagnosis at clinics, they have the option to revisit a hospital for further outpatient consultations.

^e^ In this study, the annual number of diagnostic tests performed on an outpatient consultant was assumed to be one. This assumption was based on the common diagnostic practice for migraines, where the outpatient visit pathway typically begins with an outpatient consultation, followed by diagnostic tests aimed at understanding the headaches’ cause and confirming the diagnosis, typically for active migraine symptoms within a year. These tests are generally conducted a maximum of once per patient throughout the year, regardless of the number of outpatient consultations. If the patient’s consultations continue into the following year, retesting might be necessary for diagnosis.

^f^ The clinical practice guidelines for migraine management in China [8] are generally consistent with those in Europe [9] and the U.S. [10]. Additionally, China has established guidelines that integrate traditional Chinese and Western medicine for the prevention and treatment of migraines [11]. As reported in Supplementary Material [**1**](Supplementary%20Material%201.docx), the list of medicines was validated through the validation process of the HARDSHIP healthcare utilisation questionnaire.

^g^ As reported in Supplementary Material [**1**](Supplementary%20Material%201.docx), our review identified common complementary therapies for migraines, including acupuncture, herbs, Tui Na (Chinese massage therapy), cupping, and moxibustion [12], and these therapies are easily accessible through both public and informal healthcare settings.

**References**

1. Drummond MF, Sculpher MJ, Claxton K, Stoddart GL, Torrance GW (2015) Methods for the economic evaluation of health care programmes (4th ed.). Oxford University Press, New York, United States.

2. Guizhou Provincial Bureau of Statistics (2023) 2022 Guizhou Statistical Yearbook. China Statistics Press, Guizhou, China.

3. Wei D, Loganathan T, Wong LP (2023) Employees of the banking sector in Guizhou Province in China: Prevalence of migraine, symptoms, disability and occupational risk factors. J Headache Pain 24: 52. doi:10.1186/s10194-023-01591-4.

4. World Health Organization (2015) People's Republic of China health system review.

5. Liu R, Yu S, He M, Zhao G, Yang X, Qiao X, et al (2013) Health-care utilization for primary headache disorders in China: A population-based door-to-door survey. J Headache Pain 14: 47. doi:10.1186/1129-2377-14-47.

6. Li X, Zhou J, Tan G, Wang Y, Ran L, Chen L (2012) Diagnosis and treatment status of migraine: A clinic-based study in China. J Neurol Sci 315(1): 89-92. doi:10.1016/j.jns.2011.11.021.

7. Yu S, Zhang Y, Yao Y, Cao H (2020) Migraine treatment and healthcare costs: Retrospective analysis of the China Health Insurance Research Association (CHIRA) database. J Headache Pain 21: 53. doi:10.1186/s10194-020-01117-2.

8. Chinese Neurologists Association (2022) 中国偏头痛诊治指南 (2022版). [Guidelines for the diagnosis and treatment of migraine in China (2022 Edition)]. Chinese Journal of Pain Medicine 28(12): 881-898. Retrieved from <https://kns.cnki.net/kcms2/article/abstract?v=6xaVI2TORM3eFtmmRNdT4iXgrnTEdioULPsK7k2Vgq-27rrt1GwrrA3SLnBO1ba-T-mIGxZc0n9rLodAIS_c_fp-yH04ERa9yylhL2zEQ9euT9OR2a93_o0FmIqld6PMpXSr_ViB2odZmveLFN9Bng==&uniplatform=NZKPT&language=CHS>

9. Evers S, Áfra J, Frese A, Goadsby PJ, Linde M, May A, et al (2009) EFNS guideline on the drug treatment of migraine – revised report of an EFNS task force. Eur J Neurol 16(9): 968-981. doi:10.1111/j.1468-1331.2009.02748.x.

10. Ailani J, Burch RC, Robbins MS, the Board of Directors of the American Headache Society (2021) The American Headache Society Consensus Statement: Update on integrating new migraine treatments into clinical practice. Headache 61(7): 1021-1039. doi:10.1111/head.14153.

11. Gao C-Y, Zhang Y-Y, Wu B-S, Liu Y. (2023). 中国偏头痛中西医结合防治指南（2022年）. *Chin J Integr Med, 43*(5), 517-526. Retrieved from <https://kns.cnki.net/kcms2/article/abstract?v=6xaVI2TORM0AVdwiaS35vWQyhvqHdXWBF6_fqYFp0_lINEv-NApXjzxhMBJ-4qljYSN4fhmb4ZKlwu2jRP6yyDkRtKKxRuTdxASrR7eaI8dkdSjD4Mh9NzAMmLfSzAXhJfuoe64uY2xa-jRR-jHRww==&uniplatform=NZKPT&language=CHS>

12. McQuade JL, Meng Z, Chen Z, Wei Q, Zhang Y, Bei W, et al (2012) Utilization of and Attitudes towards Traditional Chinese Medicine Therapies in a Chinese Cancer Hospital: A Survey of Patients and Physicians. Evid Based Complement Alternat Med 2012: 504507. doi:10.1155/2012/504507.
