# Supplementary material for: Healthcare utilisation and economic burden of migraines among bank employees in China: a probabilistic modelling study
Source: J Headache Pain. 2024 Apr 19;25(1):60. doi: 10.1186/s10194-024-01763-w (PMC11027248; doi:10.1186/s10194-024-01763-w)
Supplement: Supplementary file 4 — Additional file 4: Supplementary Material 4. Point estimation of the economic burden of migraines among bank employees (in 2022 USD). [file 10194_2024_1763_MOESM4_ESM.docx]

**Point estimation of the economic burden of migraines among bank employees (in 2022 USD)**

The point estimation of the economic burden of migraines was conducted based on the decision-analytic model illustrated in Fig. [2](main%20manuscript.docx) in the main manuscript. In cases where input parameters exhibited skewed distributions, median values were employed in this analysis.

In this study, the survey on healthcare utilisation related to migraines was conducted in Guizhou province, China. Accordingly, the estimation was carried out in this specific province.

1. **Number of migraine sufferers in the banking sector in Guizhou province**

Due to the unavailability of specific statistics on the population size of banking employees in China, employees in the financial sector were taken as a proxy population in this study. The banking sector in Guizhou province was estimated to have 16.4 thousand male sufferers of migraines and 18.9 thousand female sufferers (Table [1](#table1)). The sum for males and females provided a conservative estimate of the number of migraine sufferers in the banking sector in Guizhou province in China, totalling 35.3 thousand.

**Table 1** Number of migraine sufferers in the banking sector in Guizhou

| **Item** | **Proportion of males or females in the banking sector (%)** | **Prevalence rate (%)** | **No. of migraine sufferers**  **(Thousand)** |
| --- | --- | --- | --- |
| Column | (1) | (2) | (3) |
| Data source | Guizhou Provincial Bureau of Statistics [1] ^a^ | Wei et al. [2] | Calculated using Equation (1) ^b^ in the main manuscript:  130.4 ^c^× Column (1) × Column (2) |
| Male | 50.4 | 25.0 | 16.4 ^d^ |
| Female | 49.6 | 29.2 | 18.9 ^d^ |
| **Total** | | | **35.3** |

Abbreviation: No., Number.

^a^ The Bureau of Statistics is responsible for implementing censuses, managing surveys, and providing statistical information on national economic and social development in China [1].

^b^ Number of migraine sufferers by gender = Population size × Proportion of males or females × Prevalence rate of migraines by gender

^c^ Due to the unavailability of specific statistics on the population size of banking employees in China, employees in the financial sector were taken as a proxy population in this study. According to the Guizhou Provincial Bureau of Statistics [1], the financial sector employed 141.8 thousand individuals in 2022, with a male-to-female ratio of 50.4% to 49.6%.

^d^ The number of male migraine sufferers = 130.4 thousand × 50.4% × 25.0% = 16.4 thousand

The number of female migraine sufferers = 130.4 thousand × 49.6% × 29.2% = 18.9 thousand

1. **Annual healthcare utilisation related to migraines**

Based on the reported estimate of 35.3 thousand migraine sufferers in the banking sector in Guizhou province in China (as shown in Table [1](#table1)), together with the data on the proportions of outpatient consultations by facility type (shown in Table [1](main%20manuscript.docx) in the main manuscript) and the median values for the annual number of consultations for a patient by facility type obtained from our survey (2, 2.1, 2, 2, 3, and 5 for public clinics, public primary-level hospitals, public secondary-level hospitals, public tertiary-level hospitals, public Traditional Chinese Medicine (TCM) hospitals, and private facilities, respectively), the annual number of migraine-related outpatient consultations by facility type was calculated.

The results are presented in Table [2](#table2): 6.1 thousand outpatient consultations at public clinics, 3.5 thousand consultations at public primary-level hospitals, 4.7 thousand consultations at public secondary-level hospitals, 3.3 thousand consultations at public tertiary-level hospitals, 3.8 thousand consultations at public TCM hospitals, and 3.7 thousand consultations at private facilities. The sum for all facilities provided a conservative estimate of the annual number of migraine-related outpatient consultations, totalling 25.1 thousand.

Table 2 Annual number of migraine-related outpatient consultations in the banking sector in Guizhou province

| **Facility** | **Proportion of outpatient**  **consultations (%)** | **Median for the annual no. of**  **consultations for a patient** | **Annual no. of consultations**  **(Thousand)** |
| --- | --- | --- | --- |
| Column | (1) | (2) | (3) |
| Source | Table [1](main%20manuscript.docx) in the main manuscript | Supplementary Material [3](Supplementary%20Material%203.docx), source from our survey | Calculated using equation ^a^:  35.3 ^b^ ×Column (1) ×Column (2) |
| Public clinics | 8.7% | 2 | 6.1 ^c^ |
| Public primary-level hospitals | 4.7% | 2.1 | 3.5 ^c^ |
| Public secondary-level hospitals | 6.6% | 2 | 4.7 ^c^ |
| Public tertiary-level hospitals | 4.7% | 2 | 3.3 ^c^ |
| Public TCM hospitals | 3.6% | 3 | 3.8 ^c^ |
| Private facilities | 2.1% | 5 | 3.7 ^c^ |
| **Total** | | | **25.1** |

Abbreviation: TCM, Traditional Chinese Medicine; no, number.

^a^ Annual number of outpatient consultations by facility type = Number of migraine sufferers × Proportion of outpatient consultations by facility type × Annual number of consultations for a patient by facility type

^b^ The total number of migraine sufferers in the banking sector in Guizhou province, at 35.3 thousand (Table [1](#table1)).

^c^ The annual number of outpatient consultations at public clinics = 35.3 thousand × 8.7% × 2 = 6.1 thousand consultations

The annual number of outpatient consultations at public primary-level hospitals = 35.3 thousand × 4.7% × 2.1 = 3.5 thousand consultations

The annual number of outpatient consultations at public secondary-level hospitals = 35.3 thousand × 6.6% × 2 = 4.7 thousand consultations

The annual number of outpatient consultations at public tertiary-level hospitals = 35.3 thousand × 4.7% × 2 = 3.3 thousand consultations

The annual number of outpatient consultations at public TCM hospitals = 35.3 thousand × 3.6% × 3 = 3.8 thousand consultations

The annual number of outpatient consultations at private facilities = 35.3 thousand × 2.1% × 5 = 3.7 thousand consultations

Based on the reported estimate of 35.3 thousand migraine sufferers in the banking sector in Guizhou province in China (as shown in Table [1](#table1)), together with the data on the proportions of outpatient consultations by hospital type (shown in Table [1](main%20manuscript.docx) in the main manuscript) and the proportions of Computed Tomography (CT) scan, Magnetic Resonance Imaging (MRI), Transcranial Doppler ultrasonography (TCD), and electroencephalography by hospital type obtained from our survey, the annual number of migraine-related diagnostic tests by test and hospital type was calculated. The results are presented in Table [3](#table3).

Table 3 Annual number of migraine-related diagnostic tests at public facilities in the banking sector in Guizhou province

| **Row** | **Test** | **Data source** | **Primary-Level**  **hospitals** | **Secondary-Level**  **hospitals** | **Tertiary-Level**  **hospitals** | **TCM**  **hospitals** |
| --- | --- | --- | --- | --- | --- | --- |
| **CT scan** | | | | | | |
| (1) | Proportion of a scan (%) | Proportion of outpatient consultations by hospital type ^a^ × Proportion of scan by hospital type ^b^ | 3.3% | 2.9% | 2.5% | 1.4% |
| (2) | **Annual no. of CT scan (Thousand)** | Calculated using equation ^c^: 35.3 ^d^ × Row (1) | **1.2 ^e^** | **1.0 ^e^** | **0.9 ^e^** | **0.5 ^e^** |
| **MRI** | | | | | |  |
| (3) | Proportion of an MRI (%) | Proportion of outpatient consultations by hospital type ^a^ × Proportion of MRI by hospital type ^b^ | 1.0% | 1.8% | 1.5% | 1.1% |
| (4) | **Annual no. of MRI (Thousand)** | Calculated using equation ^c^: 35.3 ^d^ × Row (3) | **0.4 ^f^** | **0.6 ^f^** | **0.5 ^f^** | **0.4 ^f^** |
| **TCD** | | | | | |  |
| (5) | Proportion of a TCD (%) | Proportion of outpatient consultations by hospital type ^a^ × Proportion of TCD by hospital type ^b^ | 0.7% | 1.1% | 1.5% | 0.7% |
| (6) | **Annual no. of TCD (Thousand)** | Calculated using equation ^c^: 35.3 ^d^ × Row (5) | **0.2 ^g^** | **0.4 ^g^** | **0.5 ^g^** | **0.2 ^g^** |
| **EEG** | | | | | |  |
| (7) | Proportion of an EEG (%) | Proportion of outpatient consultations by hospital type ^a^ × Proportion of EEG by hospital type ^b^ | 0.9% | 2.6% | 2.3% | 0.7% |
| (8) | **Annual no. of EEG (Thousand)** | Calculated using equation ^c^: 35.3 ^d^ × Row (7) | **0.3 ^h^** | **0.9 ^h^** | **0.8 ^h^** | **0.2 ^h^** |

Abbreviations: TCM, Traditional Chinese Medicine; CT, Computed Tomography; MRI, Magnetic Resonance Imaging, TCD: Transcranial Doppler ultrasonography, EEG, Electroencephalography.

^a^ The proportion of outpatient consultations at public primary-level hospitals, public secondary-level hospitals, public tertiary-level hospitals and public TCM hospitals was 4.7%, 6.6%, 4.7% and 3.6%, respectively, as shown in Table [1](main%20manuscript.docx) in the main manuscript**.**

^b^ The proportion of CT scan, MRI, TCD, and electroencephalography by hospital type can be found in Supplementary Material [3](Supplementary%20Material%203.docx), sourced from our survey.

^c^ Annual number of diagnostic tests by test type and hospital type = Number of migraine sufferers × Proportion of outpatient consultations by hospital type × Proportion of diagnostic tests by test type and hospital type

According to Supplementary Material [3](Supplementary%20Material%203.docx), in this study, the annual number of diagnostic tests performed on an outpatient consultant was assumed to be one. This assumption was based on the common diagnostic practice for migraines, where the outpatient visit pathway typically begins with an outpatient consultation, followed by diagnostic tests aimed at understanding the headaches’ cause and confirming the diagnosis, typically for active migraine symptoms within a year. These tests are generally conducted a maximum of once per patient throughout the year, regardless of the number of outpatient consultations. If the patient’s consultations continue into the following year, retesting might be necessary for diagnosis.

^d^ The total number of migraine sufferers in the banking sector in Guizhou province, at 35.3 thousand (Table [1](#table1)).

^e^ Annual number of CT scans at public primary-level hospitals = 35.3 thousand × 3.3% = 1.2 thousand tests

Annual number of CT scans at public secondary-level hospitals = 35.3 thousand × 2.9% = 1.0 thousand tests

Annual number of CT scans at public tertiary-level hospitals = 35.3 thousand × 2.5% = 0.9 thousand tests

Annual number of CT scans at public TCM hospitals = 35.3 thousand × 1.4% = 0.5 thousand tests

^f^ Annual number of MRI tests at public primary-level hospitals = 35.3 thousand × 1.0% = 0.4 thousand tests

Annual number of MRI tests at public secondary-level hospitals = 35.3 thousand × 1.8% = 0.6 thousand tests

Annual number of MRI tests at public tertiary-level hospitals = 35.3 thousand × 1.5% = 0.5 thousand tests

Annual number of MRI tests at public TCM hospitals = 35.3 thousand × 1.1% = 0.4 thousand tests

^g^ Annual number of TCD tests at public primary-level hospitals = 35.3 thousand × 0.7% = 0.2 thousand tests

Annual number of TCD tests at public secondary-level hospitals = 35.3 thousand × 1.1% = 0.4 thousand tests

Annual number of TCD tests at public tertiary-level hospitals = 35.3 thousand × 1.5% = 0.5 thousand tests

Annual number of TCD tests at public TCM hospitals = 35.3 thousand × 0.7% = 0.2 thousand tests

^h^ Annual number of EEG tests at public primary-level hospitals = 35.3 thousand × 0.9% = 0.3 thousand tests

Annual number of EEG tests at public secondary-level hospitals = 35.3 thousand × 2.6% = 0.9 thousand tests

Annual number of EEG tests at public tertiary-level hospitals = 35.3 thousand × 2.3% = 0.8 thousand tests

Annual number of EEG tests at public TCM hospitals = 35.3 thousand × 0.7% = 0.2 thousand tests

Based on the reported estimate of 35.3 thousand migraine sufferers in the banking sector in Guizhou province in China (as shown in Table [1](#table1)), together with the data on the proportions of medicine use by type (Table [2](main%20manuscript.docx) in the main manuscript) and the median values for the annual days a patient was on medication by medicine type obtained from our survey, the annual medication days by medicine type was calculated. The results are presented in Table [4](#table4).

**Table 4** Annual medication days for each migraine medicine in the banking sector in Guizhou province

| **Medicine** | | **Proportion of use (%)** | **Annual days** **a patient**  **was on medication ^b^** | **Annual medication days**  **(Thousand)** |
| --- | --- | --- | --- | --- |
| Column | | (1) | (2) | (3) |
| Data source | | Table [2](main%20manuscript.docx) in the main manuscript | Our survey | Calculated using equation ^c^:  35.3 ^d^ × Column (1) × Column (2) |
| **Acute medicines** | | | | |
|  | **Traditional Chinese patent medicines ^a^** | | | |
|  | Gastrodia Capsule | 4.1% | 60.0 | 86.8 ^e^ |
|  | Zhengtian Pill | 0.9% | 195.5 | 86.8 |
|  | Tou Tongning Capsule | 2.7% | 49.1 | 62.1 |
|  | Duliang Soft Capsule | 0.5% | 36.4 | 46.9 |
|  | Yangxue Qingnao Granule | 1.1% | 46.4 | 6.4 |
|  | Lingyangjiao Pill | 0.8% | 72.0 | 18.2 |
|  | Ershiwuwei Shanhu Pill | 0.2% | 12.0 | 20.3 |
|  | Tongtian Oral Liquid | 0.8% | 124.8 | 0.8 |
|  | Tablet of Corydalistuber for Alleviating Pain | 0.9% | 30.0 | 35.2 |
|  | Seven Leaves Spirit Calmness Tablet | 0.9% | 24.0 | 9.5 |
|  | 999 Ganmaoling Granule | 0.5% | 20.4 | 7.6 |

**Table 4** continued

| **Medicine** | | **Proportion of use (%)** | **Annual days** **a patient**  **was on medication ^b^** | **Annual medication days**  **(Thousand)** |
| --- | --- | --- | --- | --- |
| Column | | (1) | (2) | (3) |
| Data source | | Table [2](main%20manuscript.docx) in the main manuscript | Our survey | Calculated using equation ^c^:  35.3 ^d^ × Column (1) × Column (2) |
|  | **Western medicines** |  |  |  |
|  | Toutong Powder | 20.4% | 24.0 | 172.8 |
|  | Aspirin | 5.5% | 36.0 | 69.9 |
|  | Ibuprofen | 26.6% | 36.0 | 338.0 |
|  | Naproxen | 0.6% | 16.8 | 3.6 |
|  | Paracetamol, aminophenazone, caffeine, and chlorphenamine maleate tablets | 3.8% | 24.0 | 32.2 |
|  | Diclofenac Sodium Sustained Release Capsule | 1.3% | 16.8 | 7.7 |
|  | Acetaminophen (Paracetamol) | 11.4% | 48.0 | 193.2 |
|  | Ibuprofen and codeine | 0.7% | 60.0 | 14.8 |
|  | Tramadol | 0.2% | 12.0 | 0.8 |
|  | Nicergoline | 0.7% | 60.0 | 14.8 |
|  | Ergotamine Tartrate/Caffeine | 0.4% | 33.4 | 4.7 |
|  | Barbiturates | 0.2% | 24.0 | 1.7 |

**Table 4** continued

| **Medicine** | | **Proportion of use (%)** | **Annual days** **a patient**  **was on medication ^b^** | **Annual medication days**  **(Thousand)** |
| --- | --- | --- | --- | --- |
| Column | | (1) | (2) | (3) |
| Data source | | Table [2](main%20manuscript.docx) in the main manuscript | Our survey | Calculated using equation ^c^:  35.3 ^d^ × Column (1) × Column (2) |
|  | Metoclopramide | 0.2% | 108.0 | 7.6 |
|  | Domperidone | 0.2% | 108.0 | 7.6 |
|  | Glucocorticoids | 0.2% | 96.0 | 6.8 |
|  | Mannitol injection | 0.2% | 60.0 | 4.2 |
|  | Sumatriptan | 0.5% | 31.1 | 5.5 |
|  | Zolmitriptan | 1.0% | 49.2 | 17.4 |
|  | Rizatriptan | 1.2% | 36.7 | 15.8 |
|  | Japan EVE QUICK Painkiller | 0.5% | 24.0 | 4.2 |
| **Preventive medicines** | | | | |
|  | Sibelium (Flunarizine) | 5.9% | 7.0 | 14.6 |
|  | Lomefloxacin | 5.5% | 7.0 | 13.6 |
|  | β1-receptor antagonists | 1.2% | 7.0 | 3.0 |
|  | Magnesium valproate | 1.4% | 7.3 | 3.6 |
|  | Sodium valproate | 1.4% | 7.7 | 3.8 |

**Table 4** continued

| **Medicine** | | **Proportion of use (%)** | **Annual days** **a patient**  **was on medication ^b^** | **Annual medication days**  **(Thousand)** |
| --- | --- | --- | --- | --- |
| Column | | (1) | (2) | (3) |
| Data source | | Table [2](main%20manuscript.docx) in the main manuscript | Our survey | Calculated using equation ^c^:  35.3 ^d^ × Column (1) × Column (2) |
|  | Topiramate | 1.0% | 7.0 | 2.5 |
|  | Gabapentin | 0.6% | 7.0 | 1.5 |
|  | Vitamin B_2_ | 3.8% | 7.0 | 9.4 |
|  | Coenzyme Q10 | 1.0% | 11.7 | 4.1 |
|  | Candesartan Cilexetil | 1.5% | 7.0 | 3.7 |
|  | Prednisone | 0.7% | 27.3 | 6.7 |
|  | Duliang Soft Capsule | 0.2% | 30.0 | 2.1 |
|  | Yangxue Qingnao Granule | 0.2% | 30.0 | 2.1 |

^a^ With the advancement of TCM, Chinese herbal tonics have evolved into what are known as traditional Chinese patent medicines. These medicines are widely employed in clinical practice in China and are available in various forms like pills, capsules, or syrups.

^b^ For acute medicines, the annual medication days for a patient = The median value of medication days reported by respondents for the last month × 12

For preventive medicines, respondents were asked directly about the number of days they used preventive medicines during the last year. The median number was used.

^c^ Annual medication days by medicine type = Number of migraine sufferers × Proportion of use by medicine type × Annual days a patient was on medication by medicine type

^d^ The total number of migraine sufferers in the banking sector in Guizhou province, at 35.3 thousand (Table [1](#table1)).

^e^ The calculation process is exemplified using Gastrodia Capsule.

The annual medication days of Gastrodia Capsule = 35.3 thousand × 4.1% × 60 days = 86.8 thousand days

Based on the reported estimate of 35.3 thousand migraine sufferers in the banking sector in Guizhou province in China (as shown in Table [1](#table1)), together with the data on the proportions of complementary therapy use by therapy and facility type obtained from our survey, the annual number of patients receiving migraine-related complementary therapies by therapy and facility type was calculated. The results are presented in Table [5](#table5): 8.4 thousand patients were estimated to use migraine-related complementary therapies at public facilities and 11.3 thousand patients at informal facilities.

**Table 5** Annual number of patients receiving migraine-related complementary therapies in the banking sector in Guizhou province

| **Complementary therapy** | **At public facilities** | |  | **At informal facilities** | |
| --- | --- | --- | --- | --- | --- |
|  | **Proportion**  **of therapies (%)** | **Annual no. of patients** **receiving complementary therapies (Thousand)** |  | **Proportion**  **of therapies (%)** | **Annual no. of patients receiving complementary therapies (Thousand)** |
| Column | (1) | (2) |  | (3) | (4) |
| Source | Supplementary Material [3](Supplementary%20Material%203.docx), sourced from our survey | Calculated using equation ^a^:  35.3 ^b^ × Column (1) |  | Supplementary Material [3](Supplementary%20Material%203.docx), sourced from our survey | Calculated using equation ^a^:  35.3 ^b^ ×Column (3) |
| Acupuncture | 7.0% | 2.5 ^c^ |  | 7.5% | 2.6 ^d^ |
| Moxibustion | 5.1% | 1.8 ^c^ |  | 5.9% | 2.1 ^d^ |
| Cupping | 3.8% | 1.3 ^c^ |  | 3.9% | 1.4 ^d^ |
| Tui Na | 3.9% | 1.4 ^c^ |  | 8.7% | 3.1 ^d^ |
| Chinese herbal medicine | 4.1% | 1.4 ^c^ |  | 3.9% | 1.4 ^d^ |
| Others | N/A | N/A |  | 1.9% | 0.7 ^d^ |
| **Total** | **N/A** | **8.4** |  | **N/A** | **11.3** |

Abbreviation: N/A, Not Available.

Note: Tui Na refers to Chinese massage therapy.

^a^ Annual number of patients receiving complementary therapies by therapy and facility type = Number of migraine sufferers × Proportion of use by therapy and facility type

^b^ The total number of migraine sufferers in the banking sector in Guizhou province, at 35.3 thousand (Table [1](#table1)).

^c^ The annual number of patients receiving acupuncture therapies at public facilities = 35.3 thousand × 7.0% = 2.5 thousand patients

The annual number of patients receiving moxibustion therapies at public facilities = 35.3 thousand × 5.1% = 1.8 thousand patients

The annual number of patients receiving cupping therapies at public facilities = 35.3 thousand × 3.8% = 1.3 thousand patients

The annual number of patients receiving Tui Na therapies at public facilities = 35.3 thousand × 3.9% = 1.4 thousand patients

The annual number of patients receiving Chinese herbal medicine therapies at public facilities = 35.3 thousand × 4.1% = 1.4 thousand patients

^d^ The annual number of patients receiving acupuncture therapies at informal facilities = 35.3 thousand × 7.5% = 2.6 thousand patients

The annual number of patients receiving moxibustion therapies at informal facilities = 35.3 thousand × 5.9% = 2.1 thousand patients

The annual number of patients receiving cupping therapies at informal facilities = 35.3 thousand × 3.9% = 1.4 thousand patients

The annual number of patients receiving Tui Na therapies at informal facilities = 35.3 thousand × 8.7% = 3.1 thousand patients

The annual number of patients receiving Chinese herbal medicine therapies at informal facilities = 35.3 thousand × 3.9% = 1.4 thousand patients

The annual number of patients receiving other therapies at informal facilities = 35.3 thousand × 1.9% = 0.7 thousand patients

1. **Annual direct costs of migraines**

The costs of outpatient visits were calculated separately for public and private facilities. Specifically, for public facilities, the costs of both outpatient consultations and diagnostic tests were combined.

Utilising the unit costs of outpatient consultations by facility type in 2022 (see Supplementary Material [2](Supplementary%20Material%202.docx)) and the annual number of consultations by public facility type (see Table [2](#table2)), the annual costs of outpatient consultations by public facility type were calculated. The results are presented in Table [6](#table6). The sum of the costs across all public facilities provided a conservative estimate of the costs for migraine-related outpatient consultations at public facilities, totalling $34.1 thousand in 2022 United States dollars (USD).

Utilising the unit costs of diagnostic tests by test and hospital type in 2022 (see Supplementary Material [2](Supplementary%20Material%202.docx)) and the annual number of these tests by test and public hospital type (see Table [3](#table3)), the annual costs of diagnostic tests by test and public hospital type were calculated. The results are presented in Table [7](#table7). The sum of the costs for all tests across all public hospitals provided a conservative estimate of the costs for migraine-related diagnostic tests at public facilities, totalling $567.6 thousand in 2022 USD.

The aggregated annual costs of outpatient consultations and diagnostic tests at public facilities amounted to $601.7 thousand.

**Table 6** Annual costs for migraine-related outpatient consultations at public facilities in the banking sector in Guizhou (in 2022 USD)

| **Facility** | **Annual no. of outpatient consultations (Thousand)** | **Unit cost (USD)** | **Annual costs for outpatient consultations (Thousand USD)** |
| --- | --- | --- | --- |
| Column | (1) | (2) | (3) |
| Data Source | Table [2](#table2) | Supplementary Material [2](Supplementary%20Material%202.docx) | Calculated using Equation (3) ^a^ in the main manuscript:  Column (1) × Column (2) |
| Public clinics | 6.1 | 0.7 | 4.3 ^b^ |
| Public primary-level hospitals | 3.5 | 1.3 | 4.6 ^b^ |
| Public secondary-level hospitals | 4.7 | 2.3 | 10.8 ^b^ |
| Public tertiary-level hospitals | 3.3 | 2.3 | 7.6 ^b^ |
| Public TCM hospitals | 3.8 | 1.8 | 6.8 ^b^ |
| **Total** | **25.1** | **N/A** | **34.1** |

Abbreviations: TCM, Traditional Chinese Medicine; USD, United States dollars; N/A, Not Applicable.

^a^ Costs for outpatient consultations or diagnostic tests = Unit cost of a consultation/test × Annual number of consultations/tests

^b^ Annual costs for outpatient consultations at public clinics = $0.7 × 6.1 thousand = $4.3 thousand

Annual costs for outpatient consultations at public primary-level hospitals = $1.3 × 3.5 thousand = $4.6 thousand

Annual costs for outpatient consultations at public secondary-level hospitals = $2.3 × 4.7 thousand = $10.8 thousand

Annual costs for outpatient consultations at public tertiary-level hospitals = $2.3 × 3.3 thousand = $7.6 thousand

Annual costs for outpatient consultations at public TCM hospitals = $1.8 × 3.8 thousand = $6.8 thousand

**Table 7** Annual costs for migraine-related diagnostic tests at public facilities in the banking sector in Guizhou province (in 2022 USD)

| **Test** | | **Annual no. of tests (Thousand)** | **Unit cost (USD)** | **Annual costs for diagnostic tests (Thousand USD)** |
| --- | --- | --- | --- | --- |
| Column | | (1) | (2) | (3) |
| Data Source | | Table [3](#table3) | Supplementary Material [2](Supplementary%20Material%202.docx) | Calculated using Equation (3) ^a^ in the main manuscript:  Column (1) × Column (2) |
| **Public primary-level hospitals** | | | | |
|  | CT scan | 1.2 | 31.3 | 37.6 ^b^ |
|  | MRI | 0.4 | 170.5 | 68.2 ^b^ |
|  | TCD | 0.2 | 22.7 | 4.5 ^b^ |
|  | Electroencephalography | 0.3 | 7.1 | 2.1 ^b^ |
| **Public secondary-level hospitals** | | | | |
|  | CT scan | 1.0 | 36.9 | 36.9 ^c^ |
|  | MRI | 0.6 | 204.5 | 122.7 ^c^ |
|  | TCD | 0.4 | 28.4 | 11.4 ^c^ |
|  | Electroencephalography | 0.9 | 8.5 | 7.7 ^c^ |
| **Public tertiary-level hospitals** | | | | |
|  | CT scan | 0.9 | 42.6 | 38.3 ^d^ |
|  | MRI | 0.5 | 227.3 | 113.7 ^d^ |
|  | TCD | 0.5 | 31.3 | 15.7 ^d^ |
|  | Electroencephalography | 0.8 | 9.9 | 7.9 ^d^ |

**Table 7** continued

| **Test** | | **Annual no. of tests (Thousand)** | **Unit cost (USD)** | **Annual costs for diagnostic tests (Thousand USD)** |
| --- | --- | --- | --- | --- |
| Column | | (1) | (2) | (3) |
| Data Source | | Table [3](#table3) | Supplementary Material [2](Supplementary%20Material%202.docx) | Calculated using Equation (2) ^a^ in the main manuscript:  Column (1) × Column (2) |
| **Public TCM hospitals** | | | | |
|  | CT scan | 0.5 | 35.0 | 17.5 ^e^ |
|  | MRI | 0.4 | 191.6 | 76.6 ^e^ |
|  | TCD | 0.2 | 26.1 | 5.2 ^e^ |
|  | Electroencephalography | 0.2 | 8.0 | 1.6 ^e^ |
| **Total** | | **9.0** | **N/A** | **567.6** |

Abbreviations: USD, United States dollars; TCM, Traditional Chinese Medicine; CT, Computed Tomography; MRI, Magnetic Resonance Imaging, TCD, Transcranial Doppler ultrasonography; N/A, Not Applicable.

^a^ Costs for outpatient consultations or diagnostic tests = Unit cost of a consultation/test × Annual number of consultations/tests

^b^ Annual costs for CT scans at public primary-level hospitals = $31.3 × 1.2 thousand = $37.6 thousand

Annual costs for MRI tests at public primary-level hospitals = $170.5 × 0.4 thousand = $68.2 thousand

Annual costs for TCD tests at public primary-level hospitals = $22.7 × 0.2 thousand = $4.5 thousand

Annual costs for electroencephalography tests at public primary-level hospitals = $7.1 × 0.3 thousand = $2.1 thousand

^c^ Annual costs for CT scans at public secondary-level hospitals = $36.9 × 1.0 thousand = $36.9 thousand

Annual costs for MRI tests at public secondary-level hospitals = $204.5 × 0.6 thousand = $122.7 thousand

Annual costs for TCD tests at public secondary-level hospitals = $28.4× 0.4 thousand = $11.4 thousand

Annual costs for electroencephalography tests at public secondary-level hospitals = $8.5 × 0.9 thousand = $7.7 thousand

^d^ Annual costs for CT scans at public tertiary-level hospitals = $42.6 × 0.9 thousand = $38.3 thousand

Annual costs for MRI tests at public tertiary-level hospitals = $227.3 × 0.5 thousand = $113.7 thousand

Annual costs for TCD tests at public tertiary-level hospitals = $31.3× 0.5 thousand = $15.7 thousand

Annual costs for electroencephalography tests at public tertiary-level hospitals = $9.9 × 0.8 thousand = $7.9 thousand

^e^ Annual costs for CT scans at public TCM hospitals = $35.0 × 0.5 thousand = $17.5 thousand

Annual costs for MRI tests at public TCM hospitals = $191.6 × 0.4 thousand = $76.6 thousand

Annual costs for TCD tests at public TCM hospitals = $26.1 × 0.2 thousand = $5.2 thousand

Annual costs for electroencephalography tests at public TCM hospitals = $8.0× 0.2 thousand = $1.6 thousand

For outpatient visits to private facilities, the costs were determined by multiplying the unit cost of $97.4 (see Supplementary Material [2](Supplementary%20Material%202.docx)) to the annual number of consultations at private facilities (see Table [2](#table2)). The calculated annual costs for outpatient visits to private facilities amounted to $360.4 thousand in 2022 USD.

The sum of the annual costs for outpatient visits to public ($601.7 thousand) and private ($360.4 thousand) facilities was the estimated annual costs for migraine-related outpatient visits, totalling 962.1 thousand.

Utilising the daily costs by medicine type (see Supplementary Material [2](Supplementary%20Material%202.docx)) and the annual medication days by medicine type (see Table [4](#table4)), the annual costs by medicine type were calculated. The results are presented in Table [8](#table8). The sum for all medicines provided a conservative estimate of the costs for migraine medicines, totalling $763.7 thousand in 2022 USD.

**Table 8** Annual costs for migraine medicines in the banking sector in Guizhou province (in 2022 USD)

| **Medicine** | | **Annual medication days (Thousand)** | **Daily cost ^b^ (CNY)** | **Annual costs for medicines**  **(Thousand CNY)** |
| --- | --- | --- | --- | --- |
| Column | | (1) | (2) | (3) |
| Data source | | Table [4](#table4) | Supplementary Material [2](Supplementary%20Material%202.docx) | Calculated using Equation (4) ^c^ in the main manuscript:  Column (1) × Column (2) |
| **Acute medicines** | | | | |
|  | **Traditional Chinese patent medicines ^a^** | | | |
|  | Gastrodia Capsule | 86.8 | 0.5 | 43.4 ^d^ |
|  | Zhengtian Pill | 62.1 | 3.9 | 242.2 |
|  | Tou Tongning Capsule | 46.9 | 6.3 | 295.5 |
|  | Duliang Soft Capsule | 6.4 | 8.1 | 51.8 |
|  | Yangxue Qingnao Granule | 18.2 | 5.1 | 92.8 |
|  | Lingyangjiao Pill | 20.3 | 7.6 | 154.3 |
|  | Ershiwuwei Shanhu Pill | 0.8 | 6.0 | 4.8 |
|  | Tongtian Oral Liquid | 35.2 | 6.8 | 239.4 |
|  | Tablet of Corydalistuber for Alleviating Pain | 9.5 | 0.7 | 6.7 |
|  | Seven Leaves Spirit Calmness Tablet | 7.6 | 0.3 | 2.3 |
|  | 999 Ganmaoling Granule | 3.6 | 1.1 | 4.0 |
|  | **Western medicines** | | | |
|  | Toutong Powder | 172.8 | 0.1 | 17.3 |
|  | Aspirin | 69.9 | 0.0 **^b^** | 1.7 |
|  | Ibuprofen | 338.0 | 0.1 | 33.8 |
|  | Naproxen | 3.6 | 0.8 | 2.9 |

**Table 8** continued

| **Medicine** | | **Annual medication days (Thousand)** | **Daily cost ^b^ (CNY)** | **Annual costs for medicines**  **(Thousand CNY)** |
| --- | --- | --- | --- | --- |
| Column | | (1) | (2) | (3) |
| Data source | | Table [4](#table4) | Supplementary Material [2](Supplementary%20Material%202.docx) | Calculated using Equation (4) ^c^ in the main manuscript:  Column (1) × Column (2) |
|  | Paracetamol, aminophenazone, caffeine, and chlorphenamine maleate tablets | 32.2 | 0.9 | 29.0 |
|  | Diclofenac Sodium Sustained Release Capsule | 7.7 | 1.0 | 7.7 |
|  | Acetaminophen (Paracetamol) | 193.2 | 0.4 | 77.3 |
|  | Ibuprofen and codeine | 14.8 | 5.7 | 84.4 |
|  | Tramadol | 0.8 | 2.6 | 2.1 |
|  | Nicergoline | 14.8 | 3.3 | 48.8 |
|  | Ergotamine Tartrate/caffeine | 4.7 | 11.6 | 54.5 |
|  | Barbiturates | 1.7 | 0.1 | 0.2 |
|  | Metoclopramide | 7.6 | 0.1 | 0.8 |
|  | Domperidone | 7.6 | 0.7 | 5.3 |
|  | Glucocorticoids | 6.8 | 0.1 | 0.7 |
|  | Mannitol injection | 4.2 | 1.4 | 5.9 |
|  | Sumatriptan | 5.5 | 21.0 | 115.5 |
|  | Zolmitriptan | 17.4 | 16.0 | 278.4 |
|  | Rizatriptan | 15.8 | 42.5 | 671.5 |
|  | Japan EVE QUICK Painkiller | 4.2 | 6.4 | 26.9 |
|  | **Subtotal of acute medicines (Thousand CNY)** | | | **2,601.9** |
|  | **Subtotal of acute medicines (Thousand USD ^e^)** | | | **739.2** |

**Table 8** continued

| **Medicine** | | **Annual medication days (Thousand)** | **Daily cost ^b^ (CNY)** | **Annual costs for medicines**  **(Thousand CNY)** |
| --- | --- | --- | --- | --- |
| Column | | (1) | (2) | (3) |
| Data source | | Table [4](#table4) | Supplementary Material [2](Supplementary%20Material%202.docx) | Calculated using Equation (4) ^c^ in the main manuscript:  Column (1) × Column (2) |
| **Preventive medicines** | | | | |
|  | Sibelium (Flunarizine) | 14.6 | 0.1 | 1.5 |
|  | Lomefloxacin | 13.6 | 2.7 | 36.7 |
|  | β1-receptor antagonists | 3.0 | 0.1 | 0.3 |
|  | Magnesium valproate | 3.6 | 1.0 | 3.6 |
|  | Sodium valproate | 3.8 | 0.4 | 1.5 |
|  | Topiramate | 2.5 | 1.3 | 3.3 |
|  | Gabapentin | 1.5 | 1.2 | 1.8 |
|  | Vitamin B_2_ | 9.4 | 0.0 **^b^** | 0.2 |
|  | Coenzyme Q10 | 4.1 | 0.6 | 2.5 |
|  | Candesartan Cilexetil | 3.7 | 1.7 | 6.3 |
|  | Prednisone | 6.7 | 0.1 | 0.7 |
|  | Duliang Soft Capsule | 2.1 | 8.1 | 17.0 |
|  | Yangxue Qingnao Granule | 2.1 | 5.1 | 10.7 |
|  | **Subtotal of preventive medicines (Thousand CNY)** | | | **86.1** |
|  | **Subtotal of preventive medicines (Thousand USD ^e^)** | | | **24.5** |
| **Total costs for acute and preventive medicines (Thousand USD)** | | | | **763.7** |

Abbreviations: CNY, Chinese yuan; USD, United States dollars.

^a^ With the advancement of TCM, Chinese herbal tonics have evolved into what are known as traditional Chinese patent medicines. These medicines are widely employed in clinical practice in China and are available in various forms like pills, capsules, or syrups.

^b^ The daily costs of Aspirin and Vitamin B2, although rounded to ¥0.0 in the data, are actually both ¥0.024.

^c^ Costs by medicine type = Daily cost by medicine type × Annual medication days by medicine type

^d^ The calculation process is exemplified using Gastrodia Capsule.

Annual costs of Gastrodia Capsule = ¥0.5 × 86.8 thousand days = ¥43.4 thousand

^e^ CNY costs were adjusted to 2022 USD using GDP deflator indexes and purchasing power parity values.

Utilising the per-patient costs for migraine-related complementary therapies in 2022 by therapy and facility type (see Supplementary Material [2](Supplementary%20Material%202.docx)) and the annual number of patients receiving these therapies by therapy and facility type (see Table [5](#table5)), the annual costs for migraine-related complementary therapies by therapy and facility type were calculated. The results are presented in Table [9](#table9). The sum of the costs for all complementary therapies at both public and informal facilities provided a conservative estimate of the annual costs for migraine-related complementary therapies, totalling $1,621.0 thousand in 2022 USD.

**Table 9** Annual costs for migraine-related complementary therapies in the banking sector in Guizhou province (in 2022 USD)

| **Complementary therapy** | **Annual no. of patients receiving complementary therapies** | **Per-patient cost**  **(USD)** | **Annual costs for complementary therapies (Thousand USD)** |
| --- | --- | --- | --- |
| Column | (1) | (2) | (3) |
| Source | Table [5](#table5) | Supplementary Material [2](Supplementary%20Material%202.docx) | Calculated using Equation (5) ^a^ in the main manuscript:  Column (1) × Column (2) |
| **At public facilities** | | | |
| Acupuncture | 2.5 | 15.4 | 38.5 ^b^ |
| Moxibustion | 1.8 | 39.6 | 71.3 ^b^ |
| Cupping | 1.3 | 14.0 | 18.2 ^b^ |
| Tui Na | 1.4 | 82.7 | 115.8 ^b^ |
| Chinese herbal medicine | 1.4 | 182.8 | 255.9 ^b^ |
| **Subtotal** | **9.3** | **N/A** | **499.7** |
| **At informal facilities** | | | |
| Acupuncture | 2.6 | 85.0 | 221.0 ^c^ |
| Moxibustion | 2.1 | 24.8 | 52.1 ^c^ |
| Cupping | 1.4 | 3.4 | 4.8 ^c^ |

**Table 9** continued

| **Complementary therapy** | **Annual no. of patients**  **receiving complementary therapies** | **Per-patient cost**  **(USD)** | **Annual costs for complementary therapies (Thousand USD)** |
| --- | --- | --- | --- |
| Column | (1) | (2) | (3) |
| Source | Table [5](#table5) | Supplementary Material [2](Supplementary%20Material%202.docx) | Calculated using Equation (5) ^a^ in the main manuscript:  Column (1) × Column (2) |
| Tui Na | 3.1 | 142.0 | 440.2 ^c^ |
| Chinese herbal medicine | 1.4 | 273.8 | 383.3 ^c^ |
| Others | 0.7 | 28.4 | 19.9 ^c^ |
| **Subtotal** | **12.2** | **N/A** | **1,121.3** |
| **Total for complementary therapies** | | | **1,621.0** |

Abbreviation: USD, United States dollars; N/A, Not Applicable.

Note: Tui Na refers to Chinese massage therapy.

^a^ Costs for complementary therapies = Per-patient cost for complementary therapies in 2022 × Annual number of patients receiving these therapies

^b^ Annual costs for acupuncture therapies at public facilities = $15.4 × 2.5 thousand patients = $38.5 thousand

Annual costs for moxibustion therapies at public facilities = $39.6 × 1.8 thousand patients = $71.3 thousand

Annual costs for cupping therapies at public facilities = $14.0 × 1.3 thousand patients = $18.2 thousand

Annual costs for Tui Na therapies at public facilities = $82.7 × 1.4 thousand patients = $115.8 thousand

Annual costs for Chinese herbal medicine therapies at public facilities = $182.8 × 1.4 thousand patients = $255.9 thousand

^c^ Annual costs for acupuncture therapies at informal facilities = $85.0 × 2.6 thousand patients = $221.0 thousand

Annual costs for moxibustion therapies at informal facilities = $24.8 × 2.1 thousand patients = $52.1 thousand

Annual costs for cupping therapies at informal facilities = $3.4 × 1.4 thousand patients = $4.8 thousand

Annual costs for Tui Na therapies at informal facilities = $142.0 × 3.1 thousand patients = $440.2 thousand

Annual costs for Chinese herbal medicine therapies at informal facilities = $273.8 × 1.4 thousand patients = $383.3 thousand

Annual costs for other therapies at informal facilities = $28.4 × 0.7 thousand patients = $19.9 thousand

Table [10](#table10) compiles the costs for outpatient visits, medicines, and complementary therapies. In 2022 USD, the annual costs included $962.1 thousand for outpatient visits, $763.7 thousand for medicines, and $1,621.0 thousand for complementary therapies. The total direct costs of migraines in the bank employee population of Guizhou province, including costs for outpatient visits, medicines, and complementary therapies, amounted to $3,346.8 thousand.

**Table 10** Annual direct costs of migraines in the banking sector in Guizhou province, China (in 2022 USD)

| **Item** | | **Data source** | **Annual costs (Thousand USD)** |
| --- | --- | --- | --- |
| **Outpatient visits** | | | |
|  | At public facilities | Section 3, Page 16-20 | 601.7 |
|  | At private facilities | Section 3, Page 21 | 360.4 |
| **Total costs for outpatient visits** | | | 962.1 |
| **Medicines** | | | |
|  | Acute medicines | Table [8](#table8) | 739.2 |
|  | Preventive medicines | Table [8](#table8) | 24.5 |
| **Total costs for medicines** | | | 763.7 |
| **Complementary therapies** | | | |
|  | At public facilities | Table [9](#table9) | 499.7 |
|  | At informal facilities | Table [9](#table9) | 1,121.3 |
| **Total costs for complementary therapies** | | | 1,621.0 |
| **Direct costs ^a^** | | | 3,346.8 |

Abbreviation: USD, United States dollars.

^a^ Calculated using [Equation (2) in the main manuscript](main%20manuscript.docx): Direct costs = Costs for outpatient visits + Costs for medicines + Costs for complementary therapies = $962.1 thousand + $763.7 thousand + $1,621.0 thousand = $3,346.8 thousand

1. Annual indirect costs of migraines

Initially, the calculation of annual lost workdays for a patient due to migraine was determined by multiplying the number of lost workdays over a three-month period (six days for male patients and two days for female patients, as derived from [our](#Table55) survey), by four, resulting in 24 days for male patients and eight days for female patients. Next, using the estimated number of migraine sufferers in the banking sector in Guizhou province (16.4 thousand males and 18.9 thousand females, as shown in Table [1](#table1)), along with the estimated daily wages of $83.9 for both genders in 2022 (refer to the [main manuscript](main%20manuscript.docx), derived from our survey), the annual indirect costs by gender were calculated. The results are presented in Table [11](#table11). The combined indirect costs for both males and females, which represented a conservative estimate of the indirect costs of migraines, amounted to $45,708.7 thousand in 2022 USD for the bank employee population of Guizhou province.

**Table 11** Annual indirect costs of migraines in the banking sector in Guizhou province, China (in 2022 USD)

| **Item** | **No. of migraine sufferers (Thousand)** | **Median lost workdays over a three-month period for a patient due to migraine** | **Annual lost workdays for a patient due to migraine** | **Annual lost workdays due to migraines** | **Median daily wage (USD)** | **Annual indirect costs**  **(Thousand USD)** |
| --- | --- | --- | --- | --- | --- | --- |
| Column | (1) | (2) | (3) | (4) | (5) | (6) |
| Data source | Table [1](#table1) | Supplementary Material [3](Supplementary%20Material%203.docx), sourced from our survey | [Column (2) × 4] ^a^ | Column (1) × Column (3) | [Main manuscript](main%20manuscript.docx)**,** calculated from our survey data | Calculated using Equation (6) ^b^ in the main manuscript:  Column (1) × Column (3) × Column (5) |
| Male | 16.4 | 6 | 24 | 393.6 | 83.9 | 33,023.0 ^c^ |
| Female | 18.9 | 2 | 8 | 151.2 | 83.9 | 12,685.7 ^c^ |
| **Total** | **35.3** | **N/A** | **N/A** | **544.8** | **N/A** | **45,708.7** |

Abbreviations: USD, United States dollars; N/A, Not Applicable.

^a^ The calculation of annual lost workdays for a patient due to migraine was determined by multiplying the number of lost workdays over a three-month period (six days for male patients and two days for female patients, as derived from our survey), by four, resulting in 24 days for male patients and eight days for female patients.

^b^ Indirect costs by gender = Number of migraine sufferers by gender × Daily wage by gender × Annual lost workdays for a patient due to migraine by gender

^c^ Annual indirect costs for male patients = 16.4 thousand patients × $83.9 × 24 days = $33,023.0 thousand

Annual indirect costs for female patients = 18.9 thousand patients × $83.9 × 8 days = $12,685.7 thousand

1. **Economic burden of migraines**

Table [12](#table12) compiles the point estimates for the annual direct and indirect costs of migraines in 2022 USD. Annually, migraines were estimated to cost the healthcare system $3,346.8 thousand, employers $45,708.7 thousand, and society $49,055.5 thousand. Notably, approximately 93.2% of the societal economic burden of migraines was attributed to the indirect costs. In the banking sector of Guizhou province in China, the societal cost per patient-year was estimated to be $1,389.7.

**Table 12** Economic burden of migraines in the banking sector in Guizhou province, China (in 2022 USD)

|  | **Direct costs** | **Indirect costs** | **Total costs** |
| --- | --- | --- | --- |
| Perspective | Healthcare system | Employers | Society |
| Data source | Table [10](#table10) | Table [11](#table11) | Direct costs + Indirect costs |
| Annual costs (Thousand USD) | 3,346.8 | 45,708.7 | 49,055.5 |
| Percentage | 6.8% | 93.2% | 100% |
| Cost per patient-year ^a^ (USD) | 94.8 | 1,294.9 | 1,389.7 |

Abbreviation: USD, United States dollars.

^a^ The total number of migraine sufferers in the banking sector in Guizhou province is 35.3 thousand (Table [1](#table1)).

Direct cost per patient-year = $3,346.8 thousand ÷ 35.3 thousand = $94.8

Indirect cost per patient-year = $45,708.7 thousand ÷ 35.3 thousand = $1,294.9

Total cost per patient-year = $49,055.5 thousand ÷ 35.3 thousand = $1,389.7

**References**

1. Guizhou Provincial Bureau of Statistics (2023) 2022 Guizhou Statistical Yearbook. China Statistics Press, Guizhou, China.

2. Wei D, Loganathan T, Wong LP (2023) Employees of the banking sector in Guizhou Province in China: Prevalence of migraine, symptoms, disability and occupational risk factors. J Headache Pain 24: 52. doi:10.1186/s10194-023-01591-4.
